# Spray-Drying Microencapsulation of Bioactive Compounds from Lemon Verbena Green Extract

**DOI:** 10.3390/foods9111547

**Published:** 2020-10-26

**Authors:** Francisco Javier Leyva-Jiménez, Jesús Lozano-Sánchez, María de la Luz Cádiz-Gurrea, Álvaro Fernández-Ochoa, David Arráez-Román, Antonio Segura-Carretero

**Affiliations:** 1 Functional Food Research and Development Center, Health Science Technological Park, Avenida del Conocimiento s/n, E-18016 Granada, Spain; jleyva@cidaf.es (F.J.L.-J.); mluzcadiz@ugr.es (M.d.l.L.C.-G.); darraez@ugr.es (D.A.-R.); ansegura@ugr.es (A.S.-C.); 2 Department of Food Science and Nutrition, University of Granada, Campus of Cartuja, 18071 Granada, Spain; 3 Department of Analytical Chemistry, Faculty of Sciences, University of Granada, Fuentenueva s/n, E-18071 Granada, Spain

**Keywords:** *Lippia citriodora*, liquid chromatography coupled to time of flight mass spectrometer (HPLC-ESI-TOF-MS), spray dry, response surface methodology, microwave-assisted extraction, antinflammatory

## Abstract

*Lippia citriodora* has been demonstrated to have a wide variety of phytochemicals which provide benefits to human health acting as antioxidants or anti-obesogenics. In this study, these phytochemicals were recovered using a microwave-assisted technology and applying optimal conditions and microencapsulated using spray drying. In this study, two different carbohydrates, maltodextrin (MD) and inulin (IN), were compared as carriers in the encapsulation procedure. The spray drying process was optimized by using a response surface methodology (RSM) based on a central composite design 2^2^, where air inlet temperature and the sample:encapsulating agent ratio (S:EA) were selected as independent variables. Both designs were analyzed equally to evaluate differences between each carrying agent on polar compounds’ encapsulation (process yield (Y%), encapsulation efficiency (EE%) and recovery of compounds (R%)) during the spray drying. The EE% and R% of each polar compound was monitored by High Performance Liquid Chromatography coupled to Time-of-Flight mass spectrometer by electrospray interface (HPLC-ESI-TOF-MS). The results showed that the use of IN as a carrier increased the powder recovered and the recovery of polar compounds after the spray dry process, whereas MD achieved a higher encapsulation efficiency.

## 1. Introduction

There is ongoing research into compounds that provide beneficial health effects. In fact, multiple beneficial properties of numerous compounds such as inulin, carotenoids or certain fatty acids have been demonstrated [1,2,3]. However, phenolic compounds, which are metabolites synthesized by plants with beneficial properties, have induced an increase in the development of foods with health promoting purposes [4,5]. For instance, *Lippia citriodora* or lemon verbena, is a plant native to South America whose phytochemicals have been demonstrated to provide antioxidant [6], anti-inflammatory [7] and anti-obesogenic effects, since they may take part in the modulation of some metabolic pathways [8,9,10]. From them, glycosylated phenylpropanoids and flavonoids have been associated to high antioxidant properties. Particularly, verbascoside and luteolin-7-diglucuronide, have exhibited great antioxidant capacities. In contrast, iridoids revealed a lower antioxidant capacity [6]. Moreover, verbascoside and martynoside, are the phenylpropanoids which have demonstrated higher modulating AMPK (adenosine monophosphate-activated protein kinase) capacity. Unexpectedly, some iridoids have been demonstrated to provide an activation of AMPK pathway [9,10].

In spite of the healthy properties of these phytochemicals, these beneficial properties have been hindered because of these compounds having several limitations that must be rectified, prior use as functional ingredients. Firstly, the concentrations available in the source, sometimes are lower than is necessary to provide beneficial effects. Moreover, the vulnerability of phenolic compounds to light, oxygen, moisture or temperature is an aspect to consider, since the oxidation of these compounds can cause a loss of their bioactivity [11]. In addition, these compounds are sensitive to changes produced throughout the gastrointestinal tract where extreme environments occur, causing changes in their structures and reducing their bioavailability as well as their bioactivity [12].

In this way, advanced extraction and encapsulation techniques are used to solve these drawbacks. This process offers a number of technological advantages such as the protection of compounds against external agents (light or humidity), masking of unpleasant characteristics or easier dispersion in the food matrix, among others [13]. In addition, these microparticles may provide a controlled release of the loads along the gastrointestinal tract, offering interesting results in in vitro and in vivo models [14,15].

Among these technologies, spray-drying is one of the most widely used in the food industry due to its low cost and flexibility. This technology consists of drying a liquid mixture composed of the core and the wall material which is atomized inside the dry spray. The water in the mixture is immediately evaporated when the drops make contact with the drying gas introduced into the equipment. The resulting microparticles (1 to 100 µm) are collected at the end of the dryer [16]. It is necessary to emphasize that the selection of the encapsulating agent is an important step in the spray dry encapsulation process since it may affect the drying process as well as the properties of the particles. For instance, maltodextrins (MD) and inulin (IN) are usually employed for microencapsulation, offering a large number of advantages [17,18].

Additionally, other parameters such as temperature of inlet drying gas or the concentration of the encapsulating agent are also important during the encapsulation process. In this sense, response surface methodologies (RSM) are used with the purpose of optimizing the encapsulation processes, reducing the costs and implementing the effectiveness of these technological processes.

The aim of this research was to develop a technological encapsulation procedure to microencapsulate antioxidant compounds of an enriched extract of *Lippia citriodora* attained by microwave assisted extraction, with the purpose of maximizing process yield, degree of encapsulation of bioactive compounds, as well as their recovery after processing using maltodextrin and inulin as the wall materials. The phytochemicals were monitored by a High Performance Liquid Chromatography coupled to Time-of-Flight mass spectrometer by electrospray interface (HPLC-ESI-TOF) analytical platform which provided individualized information about the compounds behavior during the encapsulation process. The spray dry optimization was performed applying an RSM based on a central composite design 2^2^ (CCD) model with 12 experiments where the independent variables were inlet air temperature and the sample:encapsulating agent ratio, whereas process yield, encapsulation efficiency and recovery of phytochemicals were set as the response.

## 2. Materials and Methods

### 2.1. Reagents

*L. citriodora* was kindly provided by Monteloeder (Alicante, Spain) and their leaves were milled and stored protected from the light and moisture at room temperature. The encapsulating agents, MD (20 dextrose equivalent) and IN, were purchased by Fagron (Barcelona, Spain). Standard compounds: loganic acid, verbascoside, kaempferol-3-O-glucoside, quercetin and apigenin were provided by Sigma-Aldrich (Steinheim, Germany), Extrasynthese (Genay Cedex, Francia) orFluka (Steinheim, Germany). Additionally, water was purified by a Milli-Q system from Millipore (Bedford, MA, USA) and ethanol for extraction procedure was acquired from VWR chemicals (Radnor, PA, USA). HPLC-ESI-TOF-MS analyses were performed using Liquid Chromatography- Mass Spectrometry (LC-MS) grade acetonitrile provided by Fisher chemicals (Waltham, MA, USA) whereas formic acid was provided from Sigma-Aldrich. Extraction procedures for analytical purposes were carried out using acetic acid acquired from Sigma-Aldrich and methanol LC-MS (Fisher chemicals, Waltham, MA, USA).

### 2.2. L. citriodora Extract Preparation

A Microwave assisted extraction (MAE) method was applied in order to attain an enriched extract from *L. citriodora* leaves. A Multiwave 3000 SOLV (Anton Paar, Graz, Austria) system was used in the same way that was previously described by Leyva-Jimenez et al. [19]. The conditions used were 113 °C, 42% of ethanol and 22 min of extraction with the purpose of achieving the maximum recovery of polar compounds from *L. citriodora* leaves. After the extraction procedure, the extract was homogenized, centrifuged and dried under vacuum in a Savant™ Speed Vac Concentrator SC 250 EXP (Thermo Scientific, Sunnyvale, CA, USA) and stored at −20 °C until spray drying process or HPLC-ESI-TOF/MS.

### 2.3. Microencapsulation of MAE L. Citriodora Extract by Spray Drying

The encapsulations of enriched extract of *L. citriodora* with maltodextrin and inulin were performed in accordance with an RSM based on a CCD 2^2^ model with star and four central points (Supporting Information Table S1) (Statgraphics Centurion version XVI supported by Statpoint Technologies, Warrenton, VA, USA). The independent variables were inlet air temperature (135–195 °C) and sample:encapsulating agent (S:EA) ratio (4.16–13.84) and the response variables were process yield (Y%), encapsulation efficiency of compounds (EE%) and recovery of phytochemicals after process (R%). The α values were determined according the center points of the design (4 center points). The outcomes were fitted into a second-order polynomial model as shown in Equation (1):

(1) $$Y=\;\alpha 0\;+\;\overset{k}{\underset{\;i\;=\;1}{\sum }}\alpha_{i}X_{i}+\;\overset{k}{\underset{i=1\;}{\sum }}\alpha_{ii}\;X_{i}^{2}+\;\overset{k}{\underset{i=1}{\sum }}\overset{k}{\underset{j=i+1}{\sum }}\alpha_{ij}\;X_{i}\;X_{j}$$

where Y represents the predicted response; *α*_0_ is a constant coefficient that fixed the response at the central point of the experiments, and *α_i_*¸ *α_ii_* and *α_ij_* are the regression coefficients of the linear, quadratic and interaction terms, respectively; *X_i_* and *X_j_* represent the value of independent variables. Four different parameters were used to evaluate the adequacy of the model and the adjustment of data attained: model adequacy, coefficient of determination (R^2^), lack-of-fit test and coefficient of variation (CV). All the experimental points were carried out randomly in a spray dryer 4M8-TriX (ProCept, Zelzate, Belgium) equipment comprised of a process column, an angled T transport tube, a cyclone and a product receiver.

Briefly, a mixture of *L. citriodora* extract and encapsulating agents was prepared considering 100 g of solution. In this sense, EA (2.08–6.92 g) was dissolved in distilled water (92.58–97.42 g) until a homogeneous solution was attained. It is necessary to remark that inulin was dissolved in the appropriate amount of water at 70 °C, whereas maltodextrin was dissolved at room temperature. After that, 0.5 g of *L. citriodora* extract was added and mixed by stirring until complete dilution of the extract. The resulting dilution was homogenized using an Ultra-turrax T18 (IKA-Werke, Staufen, Germany) at 15,000 RPM for 5 min. The drying procedures were carried out setting the conditions as follows: inlet air temperature, 135–195 °C; airflow, 0.30 m^3^/min; feeding flow, 2 mL/min, atomization air flow, 13 L/min; nozzle diameter, 0.6 mm; differential pressure of cyclone, 15–16 mBar whereas outlet air temperature was maintained in range of 60–90 °C. The attained microparticles after each procedure were kept protected from light and moisture at room temperature.

### 2.4. Evaluation of Variable Responses

#### 2.4.1. Process Yield Assessment (Y%)

The yield of each process was determined by considering the number of solids fed into the spray dry equipment and the powder attained at the end of the technological process. The results were calculated according to Equation (2):

(2) $$Y\;\%=\frac{Powder\;after\;spray\;dry}{Solids\;introduced\;in\;the\;feeding}\times \;100$$

#### 2.4.2. Encapsulation Efficiency Assessment (EE%)

The encapsulation efficiency was calculated by taking the non-encapsulated compounds and encapsulated compounds into account. To determine the non-encapsulated compounds, 150 mg of microparticles were dispersed in 1 mL of methanol:ethanol (50:50 *v*:*v*) and gently moved to achieve complete dispersion of particles. Then the particles were centrifuged at 1000 RPM at 4 °C for 1 min and the supernatant was recovered and then centrifuged at 2000 RPM at 4 °C for 2 min. Finally, the supernatants were collected and filtered through 0.2 µm PTFE filter. When inulin was used as the encapsulating agent, the encapsulated compounds were determined using the resulting pellet and diluting it in 0.75 mL of water:acetic acid (99:1 *v*/*v*). After 1 min in the vortex, the microparticles were sonicated for 20 min and then centrifuged at 13,000 RPM at 4 °C for 5 min. The supernatants were recovered and stored. The procedure was repeated once adding 0.75 mL of the mix to the resulting pellet. The supernatants were mixed and filtered through a 0.2 µm cellulose filter. In the case of particles with maltodextrin, a total volume of 1.5 mL was added to the pellet at the beginning of the process. After that, both supernatants were injected into HPLC-ESI-TOF/MS in order to determine the individual degree of encapsulation.

The EE% was determined using the Equation (3):

(3) $$EE\%=\frac{Total\;compound\;-Non\;encapsulated\;compound\;}{Total\;compound\;}\times \;100$$

where the total compound content is a sum of non-encapsulated compounds and encapsulated compounds.

#### 2.4.3. Recovery of Compounds after Spray Drying Process (R%)

The recovery of phytochemicals after the spray drying process was used to determine the degree of phytochemical loss during the technological procedure. This response variable was calculated considering the total amounts of individual compounds fed into the equipment and compared to the amounts recovered after the spray dry procedure. The outcomes were determined in accordance with Equation (4):

(4) $$R\%=\frac{Compounds\;recovered\;after\;spray\;dry}{Compounds\;indroduced\;in\;the\;feeding}\times 100$$

### 2.5. Evaluation of Polar Compounds in L. Citriodora Microparticles by HPLC-ESI-TOF/MS

The phytochemical composition of free extract together with encapsulated and non-encapsulated samples were assessed by HPLC-ESI-TOF/MS according to Leyva-Jimenez et al. [20] using a RRLC 1200 series (Agilent Technologies, Palo Alto, CA, USA), equipped with a vacuum degasser, an autosampler, a binary pump and a diode array detector (DAD) detector. The chromatographic separation was carried out by reverse phase and performed in a Zorbax Eclipse Plus C18 analytical column (150mm × 4.6mm id, 1.8 µm) acquired from Agilent Technologies (Palo Alto, CA, USA). The mobile phases were water:acetonitrile (90:10 *v*/*v*) with 0.1% formic acid as eluent A and acetonitrile as eluent B which were pumped following the multistep gradient previously reported [20]. A total of 10 µL of extract were injected and the compounds were separated for 35 min at room temperature. The HPLC flow rate was 0.5 mL/min. Hence, a “T” type splitter (split ratio = 1:3) was used for coupling with a time of flight mass spectrometer (microTOF, Bruker Daltonik, Bremen, Germany).

A mass spectrometer was equipped with an orthogonal electrospray interface (ESI) (model G1607 from Agilent Technologies) working in negative ionization mode and the mass range was set from 50 to 1000 *m*/*z*. All sources and transfer parameters were set in accordance with a validated previous method [20]. Each analysis was externally calibrated with a sodium formate cluster prior to each injection.

The quantification of phytochemicals contained in the extract and microparticles were also carried out by HPLC-ESI-TOF-MS. Therefore, the free extract was prepared at 5 mg/mL^−1^, by diluting the appropriate amount of extract with water:ethanol (50:50 *v*:*v*) and filtered previous injection. Four calibration curves were prepared (quercetin, verbascoside, kaempferol-3-glucoside and loganic acid) to discern the amounts of each phytochemical. To that end, twelve points at different concentrations were performed and the quantity of each compound was determined by drawing up the standard concentration as a function of the peak area (area standard/area internal standard), since apigenin was used as an internal standard in each analysis.

## 3. Results

### 3.1. Quantitative Characterization of Phytochemical by HPLC-ESI-TOF-MS

The extract of *L. citriodora* was attained by an advanced extraction system following the previously optimized conditions in order to recover the maximum amount of polar compounds from its leaves. The phytochemical composition was characterized qualitatively and quantitatively by HPLC-ESI-TOF-MS. SI Table S2 displays an overview of the chemical composition where retention time (min), *m*/*z*, molecular formula (M-H), proposed name and their quantities expressed as µg of analyte per g of extract are shown. In addition, a classification according its chemical structure was also demonstrated.

A total of 49 compounds were detected. They were classified into five different groups: organic acids, iridoids glycosides, flavonoids, phenylpropanoids and others. In the end, it was not possible to identify seven compounds with the analytical platforms applied.

A total of 267,384 µg of polar compounds per g of extract was quantified. Considering the contribution, iridoids was the lowest abundant chemical group (7118 µg of iridoids/g of extract). Theveside (peak 10), gardoside (peak two) and ixoside (peak four) were the most abundant iridoids glycosides in the extract (above 1000 µg of compound/g of extract). Nevertheless, other minor compounds belonging to this chemical group were also found. For instance, shanzhiside (peak three), myxospyroside (peak 11) and durantoside I (peak 36) were also quantified (~150 µg of compound/g of extract). The rest of these compounds: teucardoside (peak 12), lippioside II (peak 20), lippioside I and its derivative (peaks 25 and 24, respectively), hydroxycapsiside (peak 34) and lippianoside B (peak 33) were found in concentrations lower than 90 µg of compound/g of extract. Finally, manuleoside H (peak 45) was shown to have the lowest amounts of this chemical group.

Flavonoids were composed of seven compounds contributing to a total content of 13,449 µg of flavonoids/g of extract. As expected, chrysoeriol-7-diglucuronide (peak 27) presented the highest concentration with 5632 µg of compound/g of extract. On the other hand, luteolin-7-diglucuronid, dimethyl quercetin and acacetin-7-diglucuronide (peak 18, 49 and 38, respectively) showed approximately the same concentrations (~2200 µg of compound/g of extract). Lastly, apigenin-7-diglucuronide, dimethyl kaempferol and methyl quercetin (peaks, 23, 48 and 46) were detected in small amounts.

The last chemical group quantified was the phenylpropanoids which revealed to be the most abundant group in the variety of compounds, as well as concentrations (246,817 µg of phenylpropanods/g of extract). Predictably, verbascoside (peak 28) and isoverbascoside (peak 31) were the most abundant compounds, not only in this group but also in the whole extract assuming 173,472 and 54,316 µg of compound/g of extract, respectively. In addition, compounds like leucoseptoside A (peak 37), verbasoside (peak five) and martynoside or isomer (peak 42) revealed amounts higher than 2000 µg of compound/g of extract. Other compounds which showed relevant amounts in the extract were cistanoside F (peak six), β-hydroxyverbascoside and β-hydroxyisoverbascoside (peaks 17 and 19, respectively), lariciresinol glucopyranoside (peak 29) and osmanthisude B (peak 44) which were found in concentrations between 1000 and 2000 µg of compound/g of extract. Moreover, compounds verbascoside A (peak 30), forsythoside A (peak 32) and martynoside or isomer (peak 43) revealed amounts comprised between 700 and 900 µg of compound/g of extract. Finally, β-hydroxyverbascoside derivatives (peak 15 and 16), oxoverbascoside (peak 22), campenoside I (peak 26) and lipedoside A I (peak 35) assumed less than 500 µg of compound/g of extract presenting the lowest amounts in this chemical group.

On the other hand, peak one was identified as an organic acid, specifically, gluconic acid. Furthermore, compound eight was identified as primeverin presenting a retention time at 5.3 min. The next eluted compound (peak nine) was characterized as pyrocatechol glucuronide. Finally, compound 13 with a molecular formula C_18_H_28_O_9_ was associated with tuberonic acid glucoside.

### 3.2. RSM Analysis of Spray Drying Process

**Figure 1:**
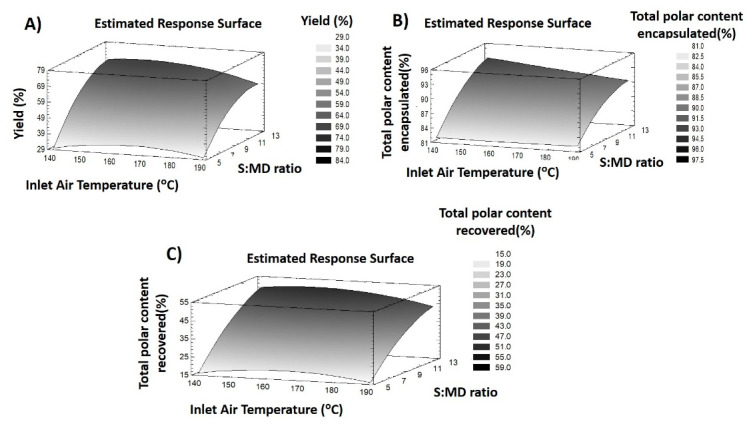
Response surface methodology (RSM) plots of responses of maltodextrin (MD) particles: (**A**) Yield, (**B**) Total polar encapsulation and (**C**) Total polar recovery. Sample:Maltodextrin (S:MD)

**Figure 2:**
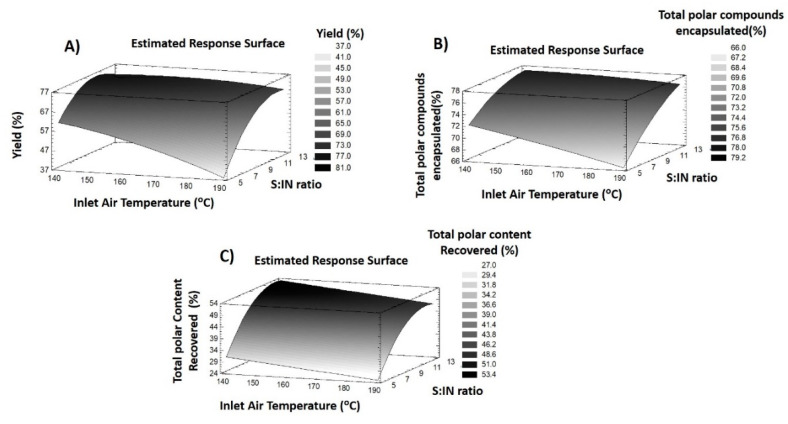
RSM plots of responses of inulin (IN) particles: **(A**) Yield, (**B**) Total polar encapsulation and (**C**) Total polar recovery. Sample:Inulin (S:IN)

With the purpose of optimizing the encapsulation process of polar compounds contained in *L. citriodora* extract using MD and IN as wall materials, two RSM experimental designs were performed to maximize the Y% as well as the EE% and R% of total polar compounds present in the extract by spray drying. Table 1 and Table 2 display the response variables and statistical results, respectively, when MD was used whereas Table 3 and Table 4 show the response variables and statistical results, respectively, when IN was used. At the same time, several analyses of variance (ANOVA) were performed in order to discern the influence of each factor during the encapsulation procedure. Moreover, the model fitting was evaluated using four different parameters. In this way, the coefficient of determination (R^2^), revealed the degree of data variations with regard to the mathematical model of the design, presenting good results when over 80%. Lack-of-fit tests were completed in order to verify the fitting quality of the model [21]. The fitting is accepted when *p*-value > 0.05. Furthermore, another parameter, called the model adequacy, exists to verify the good fitting of the model, showing good outcomes when *p* value ≤ 0.05 [22]. Lastly, the variation coefficient is used to determine the reproducibility and reliability of experiments in the investigated model with regards to the theoretical values proposed by the model. In this sense, values lower than 10% indicate a good reproducibility whereas values of between 10 and 20% were considered as acceptable [23]. These results are shown in Table 2 and Table 4. Furthermore, the experimental results were fitted into a quadratic polynomial model based on Equation (1) where the regression coefficients were established by using the method of least squares (MLS). These equations together with the response surface plots enable the understanding of the response variables’ behavior during the experimental range (Figure 1 and Figure 2).

#### 3.2.1. Response Variables of MD Particles

##### Process Yield

In order to evaluate the model fitting, the influence of each factor (inlet air temperature and S:EA ratio) and the optimum conditions for the process yield, different statistical assays were evaluated. The statistical results for MD particles are shown in Table 2. As Table 1 displays, the amounts of powder retrieved at the end of the spray dry procedure ranged from 26.10% (run 9) to 66.86% (run 3). This design revealed a relatively good fitting since the parameters model adequacy (*p* value ≤ 0.05), R^2^ (0.94) and CV (<15%) displayed acceptable values. Nevertheless, lack-of-fit test gave significant values (*p* < 0.05), revealing a deviation of the fitting.

Regarding the results of the ANOVA tests, the encapsulation of polar compounds from *L. citriodora* using MD was significantly influenced by linear and quadratic effects of temperature and the S:MD ratio. On the other hand, the interaction between the two factors did not present a significant effect. The reduced model that explains the process yield when MD was used is exposed in Equation (5). It is necessary to remark that non-significant effects were removed from the equation.

(5) $$Y{\%}_{MD}=\;-210.859+2.386\;X_{1}+13.871\;X_{2}-0.008\;X_{1}^{2}-0.532\;X_{2}^{2}$$

It can be seen that the S:MD ratio had a bigger influence on the yield than temperature, according to coefficients in Equation (5). This fact is also confirmed by the *p*-values shown in Table 2. Therefore, the process yield increased when the MD concentration in the mix fed was raised. According to Figure 1A, the increase in temperature (135 to 165 °C) did not result in important changes, although a higher increase in temperature caused a decrease in the powder collected. This result may be explained by the increase in total solid content in the mix fed and the reduction in the stickiness of the extract induced by the increase in MD concentration [24].

In spite of the fact that lack of fit test showed a deviation of this response, the rest of parameters, such as R^2^ or model adequacy allowed for a prediction of the results obtained. For this reason, an optimization of conditions to maximize the powder recovered after spray drying process was suggested. The optimum conditions would be: air inlet temperature 159 °C and an S:MD ratio of 13. The application of these drying conditions gave a theoretical yield of 70%.

##### Encapsulation of Total Polar Compounds

The EE% of total polar compounds was also evaluated. This response was assessed taking the encapsulation degree of individual compounds into account (SI Table S3). The outcomes of EE% varied from 80.74% (run nine) to 92.51% (run three). Regarding the values shown in Table 2, the encapsulation of total polar compounds revealed a good fit since the model adequacy was significant (*p* < 0.05), R^2^ was 0.96 and the CV was below 1%. Nevertheless, there was a significant result (*p* < 0.05) in lack-of-fit test. The equation describing the effects of each factor on this response is displayed in Equation (6):

(6) $$EE{\%}_{MD}=61.830+0.038X_{1}+4.551\;X_{2}-0.007\;X_{1}X_{2}-0.117X_{2}^{2}$$

As well as yield, the encapsulation of compounds was mostly affected by the linear and quadratic effects of the S:MD ratio. Although the linear effects of inlet temperature and the interaction of both factors also had a significant influence. In this sense, a graphical explanation of this response behavior is shown in Figure 1B. The increase in EE% was positively affected by increasing the concentration of MD, but the negative effect of temperature induced a reduction in the encapsulation degree. These outcomes are related to the amounts of MD available in the mix fed that allowed the encapsulation of compounds [25]. On the other hand, the application of a higher temperature might decrease the retention of polar compounds in microparticles. Similar results were found during the encapsulation of colorants in MD microparticles [26,27]. Hence, the combination of high temperatures and a low S:MD ratio will provide a smaller EE%, as shown by conditions nine and six, whereas the lower EE% of condition 11 was conditioned by the lower MD concentration used.

Despite the lack of fit due to the statistical results obtained, the optimum conditions suggested by the model, which would maximize the EE% of the total polar content, were found at a minimum air inlet temperature (134.75 °C) and a maximum S:MD ratio (13.84). These conditions revealed a theoretical value of 94% of encapsulation.

On the other hand, the lack-of-fit previously displayed by this response might be associated with an imbalance of some chemical groups towards the proposed model. In this scenario, the three different chemical groups were individually analyzed in order to discern the cause of this deviation. The results shown in Table 2 reveal that only iridoids glycosides had a good fit whereas the flavonoids and phenylpropanoids groups displayed unacceptable fitting parameters. In this sense, the results shown by the flavonoids may be explained due to the chemical structure of the compounds that belong to this chemical group—since there were flavonoids with glucuronic moieties, but also aglycones. As can be seen in Table S3, the high encapsulation efficiency revealed by diglucuronic flavonoids compared with aglycones, may occur due to the higher possibility of creating hydrogen bound between MD and diglucuronic flavonoids, compared with aglycones [28]. In the same case, the phenylpropanoids group presented a wide variety of compounds which present different behaviors in the encapsulation efficiency.

##### Recovery of Total Polar Compounds

Additionally, the recovery of compounds after the spray drying process was evaluated through the analysis of individual compound retention (SI Table S4). Overall, values below 50% were attained, showing a degradation of compounds and/or losses during the process (Table 1). Specifically, recovery of compounds was comprised from 13.42 (run nine) to 50.31% (run one). The fitting of the model for this response was also assessed and outcomes revealed a great adjustment since model adequacy was significant, R^2^ was 0.97 and the CV was below 12%. In addition, the lack-of-fit test showed a non-significant value (*p* > 0.05). Hence, the model was completely fitted to the recovery of polar compounds. The equation that summarizes the effects of each factor on this response is the following (Equation (7)):

(7) $$R{\%}_{MD}=\;-176.758+1.983\;X_{1}+8.954\;X_{2}-0.006\;X_{1}^{2}-0.283\;X_{2}^{2}$$

According to these coefficients, the S:MD ratio (linear and quadratic effects) was the factor with the highest influence on the recovery, exhibiting greater values when the MD concentration was increased. On the other hand, the effect of air inlet temperature caused an increase in recovery at the middle values (Figure 1C). The relationship between air inlet temperature and recovery of compounds at the end of the process may be associated with two conditions. (1) The lower temperatures provided a raised moisture in the MD particles, enabling its adhesion to the drying walls and (2) the higher temperatures induced a degradation of compounds [27,29]. For these reasons, middle values offered enough heat and mass transfer to provide the correct encapsulation process. Therefore, the lower values of conditions six (18.54%) and nine (13.42%) may be associated with a combination of two conditions, a reduced MD concentration that provided a low encapsulation and consequently, a higher amount of free compounds that are degraded by high temperatures. On the other hand, the lower R% of condition 11 was linked to drying chamber losses caused by the lower MD concentration fed in the instrument (S:EA 5) and lower inlet temperature (140 °C) that induced a stickiness of the particles and free extract on the walls.

In accordance with the aforementioned data, an optimization of R% was performed and proposed by the model. The suggested conditions were 155 °C of air inlet temperature and a 13.84 S:MD ratio, achieving a theoretical value of 52% recovery.

In addition, a detailed analysis of each chemical group was performed. The iridoids and phenylpropanoids revealed great fitting parameters. Nevertheless, despite exhibiting a good model adequacy, R^2^ and CV, the lack of fit test was significant, revealing any bad adjustment of the flavonoids (Table 2). These results, could be explained by the behavior of flavonoids during the encapsulation process. In this sense, according to other studies, higher temperatures may cause a thermal degradation of flavonoids with glucuronic moieties that may cause an increase in their losses along the spray drying equipment [30].

#### 3.2.2. Response Variables of IN Particles

##### Process Yield

In order to compare the spray dry process, IN was also used as an encapsulating agent. The same experimental design was applied with the purpose of assessing the variations during the drying process when this carrier was used. In this scenario, the results attained after performing the experimental design are shown in Table 3, whereas statistical assays are in Table 4. Finally, the RSM plots which summarize the behavior of each response are displayed in Figure 2.

The powder recovered after the spray drying procedure ranged from 28.51 (Run 6) to 74.70% (run 8). The statistical parameter used to verify the model adjustment, revealed an acceptable fitting. In this sense, model adequacy was significant (*p* < 0.05), a R^2^ of 0.83. The CV was acceptable since it was below 20%. However, the lack-of-fit showed a small imbalance due to it revealing a significant *p*-value. The ANOVA analyses established that all independent variables had a significant effect on the process yield, except for the quadratic effect of temperature. Equation (8) compiled the reduced model behavior of this response:

(8) $$Y{\%}_{IN}=120.410-0.747X_{1}+4.608X_{2}+0.056X_{1}^{2}-\;0.623X_{2}^{2}$$

As Figure 2A shows, the combined effects of lower inlet temperature, as well as IN concentration, caused a decrease in the powder recovered, which may explain the lower results reached in run six (28.51%). In contrast, lower temperatures and middle IN concentrations provided a great process yield, as presented in run eight (74.70%). In addition, run one, which was performed with the highest IN concentration, did not exhibit the highest yield (70.07%). It is necessary to remark, that IN addition into a solution increases the viscosity of the mixture. This may produce more solids to paste on the drying chamber wall, and consequently, reduce the total powder recovered [31].

According to the deviation of this response to the model as lack of fit test showed, a theoretical optimization was suggested considering the acceptable results obtained the rest of fitting parameters. For this purpose, the minimum temperature (134.75 °C) as well as middle concentrations of IN (S:IN ratio of 9.7) are necessary to achieve a yield of 79%.

##### Encapsulation of Total Polar Compounds

The encapsulation degree of the compounds contained in *L. citriodora* extract was also assessed by individual analysis of EE% of polar compounds (SI Table S5). EE% above 67% was achieved, with run six (67%) and run three (77%) having the lowest and the highest values reached, respectively. Regarding the adjustment of the model, all fitting parameters exhibited good values, confirming the adequacy of the model for EE% of polar compounds when IN was used as an encapsulating agent (Table 4).

With the purpose of determining which factors had a notable influence in the encapsulation process, an assessment of the reduced model equation (Equation (9)), as well as RSM plots was made (Figure 2B).

(9) $$EE{\%}_{IN}=89.222-0.178X_{1}+0.203\;X_{2}+0.012\;X_{1}X_{2}-0.071\;X_{2}^{2}$$

This equation shown together with Table 4 shows the great effect of the S:IN ratio, exhibiting a greater EE% when it was higher (run one and three). The negative effect of air inlet temperature showed that run 10 revealed a slight decrease. In the case of conditions six and nine, the combination of a higher inlet temperature and middle-lower IN concentration induced a shrinkage of the EE% of compounds (~67%). This reduction in EE% could be induced by an increase in the stickiness of the mixture, which might be caused by a reduced S:IN ratio that highlights the presence of higher amounts of non-encapsulated extract droplets in the dry chamber. This fact resulted in the augmented losses in the dry chamber wall. Moreover, the narrow quantities of IN available, limited the encapsulation of compounds. These conditions were also reported during the spray drying process of different juices [27,32]. These facts may be verified since the interaction effect of air inlet temperature and S:IN ratio also presented a significant influence.

Finally, the optimization of the EE% for total polar compounds were performed, since the fitting results allowed a reliable reproducibility of the results. In this sense, the application of lower temperature (134.75 °C) and a high S:IN ratio (13.2) will enable the attainment of 77.49%. This theoretical value is close to being reached during the performance of run three. These results are caused by the similar experimental conditions.

Additionally, an individual analysis of chemical groups was performed. The statistical outcomes shown in Table 4 exhibited a great fit for iridoids and phenylpropanoids. However, the non-significant adequacy model in the flavonoids group (*p* > 0.05) indicated an inadequate fit of these phytochemicals to the model which may be induced due to the different behavior of compounds. In this sense, diglucuronic derivatives achieved an EE% above 95% in all experimental points, whereas methylated aglycons were below 60%.

##### Recovery of Total Polar Compounds

The effects of IN addition during the encapsulation process of polar compounds from *L. citriodora* were examined regarding the results shown in Table 3 and Table 4 and the individual compound recoveries showed in SI Table S6. In this sense, the lowest recovery was reached in run six (16.82%) and the highest recovery was 52% (run three). In spite of the CV being high (below 15%), it was within an acceptable range. On the other hand, model adequacy, lack-of-fit test and R^2^ showed a great fitting of the recovery of polar compounds when IN was used. Regarding the ANOVA results, a reduced equation model, maintaining the significant factors was proposed (Equation (10)):

(10) $$R{\%}_{IN}=\;-17.164+11.363\;X_{2}-0.475\;X_{2}^{2}$$

Therefore, only the linear and quadratic effect of the S:IN ratio exhibited a significant influence. For this reason, conditions with middle and higher IN concentrations achieved greater recoveries. In addition, it can be seen that the linear effect of temperature had a marginally significant negative value (*p* < 0.1). This result may explain the behavior of runs six (~17%) and 11 (~32%), where only the air inlet temperature was changed (190 °C and 140 °C) and the R% was practically double the amount. The high concentration of inulin allowed an adequate encapsulation of compounds, increasing their protection against higher temperatures. In the same way, lower IN concentrations achieved a reduced coating of compounds which were more exposed to temperature, inducing their degradation. This behavior may be graphically seen in Figure 2C. For this reason, the optimum air inlet temperature proposed for increasing this response was 134.75 °C and S:IN ratio of 12 with a theoretical optimum value of 55%.

As well as EE% of inulin particles, a similar result was shown by individual chemical groups analyzed. In this sense, only flavonoids revealed a slight deviation due to the significant value of lack-of-fit test.

### 3.3. Comparative Evaluation of Effects Induced by Encapsulation Agents

One of the aims of this study was to evaluate the differences induced by MS and IN during the spray draying process, hence a comparative assessment of experimental results of each response was performed. Firstly, a higher recovery of powder after the drying process was achieved with IN (Table 1 and Table 2). This behavior may be caused by different glass transition temperatures (T_g_) of each carrier. T_g_ is the temperature below which the physical properties of plastics change to those of glassy or crystalline state. Therefore, an increase above the T_g_ induces a sticky behavior and consequently an enhancement of the drying chamber losses [33]. It has been demonstrated that T_g_ is influenced by the composition and the chain length of carrier polymers as well as the higher glassy transition temperatures of IN with regards to MD [34]. Because of the type of MD used, which has an elevated dextrose equivalent and consequently a T_g_ lower, induced a decreased powder retrieval when high temperatures were applied [35,36].

Concerning the EE%, MD exhibited higher skills in encapsulating total polar compounds contained in *L. citriodora* extract. Although the EE% of the whole chemical group was higher than 80%, the EE% of iridoids was markedly higher (above 90%). Conversely, flavonoids were the chemical group with a higher EE% when IN was used (above 82%). The interactions between the polymer and compounds were determinant in these results since they were related to the ability to create bonds that enable the retention of compounds. In this sense, the dextrose equivalent of MD used played an important role, since according to other studies, higher retention of compounds may be achieved when increasing the DE [37]. This increased retention may be caused by MD:polar compound interactions which could be associated with the high number of hydroxyl groups available in MD. This allowed the formation of hydrogen bonds with polar compounds shown in the extract to increase the encapsulation degree [28]. Similar results were achieved during the spray drying encapsulation of *Opuntia ficus* [38].

The recovery of total polar compounds was below 50% in both cases, indicating that the drying temperature and the encapsulating agents had a relevant effect on compound recovery. As well as yield, greater recoveries were achieved during encapsulation with IN, with iridoid glycosides and phenylpropanoids being the most recovered. However, MD microparticles allowed a higher R% of iridoids and flavonoids which may be caused by more intense interactions between these compounds with MD than with IN, which allowed a better protection against heat. In any case, a deep degradation of compounds during the procedure took place due to the use of high air inlet temperatures.

## 4. Conclusions

Two spray drying encapsulation procedures were implemented to maximize the Y%, EE% and R% of polar compounds contained in *L. citriodora* using MD and IN as encapsulating agents. Additionally, each chemical group contained in the extract was evaluated with the purpose of determining the fit towards the proposed model. The statistical analysis of the model revealed that Y% presented a slight deviation from the fitting to the proposed model during MD and IN encapsulation. EE% of total polar content showed a minor deviation when MD was used. This result could be explained after the individual fit of chemical groups present in *L. citriodora* extract, revealing that flavonoids, due to the different structures of these phytochemicals and their interactions with the MD or IN, presented a deviation in the fitting. The R% of polar compounds showed a great fit for two encapsulating agents. Furthermore, the influence of air inlet temperature and S:EA ratio was also assessed, being relevant factors in the encapsulation process by spray drying. Overall, the S:EA ratio had a relevant influence for all responses evaluated, whereas temperature had an important effect on yield and recovery of compounds, being determinant when MD was used. The individual analysis of each polar compound by HPLC-ESI-TOF-MS allowed a better understanding of the response variables’ behavior. Moreover, the theoretical values proposed by the model offered improvements compared to experimental conditions, except for the EE% of polar compounds of IN particles, since the optimum conditions were very similar to those applied in the experimental model. All outcomes proved that spray drying is an interesting encapsulation technique whose results are influenced by the air inlet temperature and the nature of the carrier selected. In addition, these microparticles may be used as functional ingredients enriched in bioactive compounds from *L. citriodora* as well as increasing the nutritional value of functional foods.

## Figures and Tables

**Table 1:** Responses of spray drying process in MD particles.

Encapsulation Conditions	Total Polar Content			Iridoids			Flavonoids			Phenylprop			Yield
	TC	EE	R	TC	EE	R	TC	EE	R	TC	EE	R	
1	133,745 ± 11,561	91.96	50.31	3626 ± 38	94.82	58.71	6719 ± 225	86.77	52.30	1,234,000 ± 1298	92.20	49.41	65.36
2		88.47	37.90		93.86	44.59		86.83	39.69		88.29	37.18	58.10
3		92.51	47.98		95.68	61.95		88.80	51.60		92.83	46.48	66.86
4		89.04	40.70		94.25	48.85		88.44	41.74		88.69	39.99	61.27
5		89.00	41.55		94.41	50.66		89.07	43.07		88.52	40.69	61.32
6		82.56	18.54		91.65	21.99		86.98	17.87		81.18	18.40	33.74
7		88.17	39.22		94.27	48.36		88.41	40.65		87.61	38.38	60.86
8		91.19	38.45		94.44	49.43		87.93	42.92		91.45	37.04	60.31
9		80.47	13,42		90.49	16.19		87.10	12.69		78.70	13.34	26.10
10		91.41	46.93		94.77	59.84		88.95	50.95		91.51	45.44	64.94
11		80.75	14.26		90.55	16.96		86.21	13.97		79.13	14.11	26.41
12		86.67	27.23		93.31	36.79		87.79	28.45		85.85	26.39	42.31

**TC:** Total compounds content (Value = X ± SD. µg of analyte/g of microparticle); **EE**: Encapsulation Efficiency; **R**: Recovery.

**Table 2:** Analysis of variance (ANOVA) of the regression models. Response variables: Yield, encapsulation and recovery of total polar content and both chemical group in Maltodextrin microparticles.

Source		Encapsulation				Recovery			
	Yield	Total Polar Content	Iridoids	Flavonoids	Phenylprop	Total Polar Content	Iridoids	Flavonoids	Phenylprop
	*p*-Value	*p*-Value	*p*-Value	*p*-Value	*p*-Value	*p*-Value	*p*-Value	*p*-Value	*p*-Value
**Model**	0.001 ^b^	0.000 ^b^	0.000 ^b^	0.278	0.000 ^b^	0.000 ^b^	0.000 ^b^	0.000 ^b^	0.000 ^b^
**X_1_:Temperature**	0.027 ^b^	0.022 ^b^	0.160	0.785	0.017 ^b^	0.093	0.162	0.034 ^b^	0.104
**X_2_: S:EA ratio**	0.000 ^b^	0.000 ^b^	0.000 ^b^	0.196	0.000 ^b^	0.000 ^b^	0.000 ^b^	0.000 ^b^	0.000 ^b^
**X_1_ X_2_**	0.057	0.039 ^b^	0.025 ^b^	0.766	0.038 ^b^	0.197	0.256	0.216	0.191
$X_{1}^{2}$	0.008 ^b^	0.751	0.429	0.962	0.745	0.016 ^b^	0.155	0.024 ^b^	0.013 ^b^
$X_{2}^{2}$	0.001 ^b^	0.003 ^b^	0.004 ^b^	0.278	0.003 ^b^	0.010 ^b^	0.015 ^b^	0.005 ^b^	0.011 ^b^
**Lack-of-fit**	0.015 ^b^	0.040 ^b^	0.193	0.429	0.023 ^b^	0.069	0.126	0.029 ^b^	0.071
**R^2^**	0.94	0.96	0.98	0.41	0.96	0.97	0.96	0.96	0.97
**CV**	<15	<1	<0.3	<1	<2	<12	<10	<12	<13

**R^2^** = Quadratic correlation coefficient; **CV** = coefficient of variation (%). ^b^ Significant (*p <* 0.050); **X_1_:** temperature; **X_2_:** Sample-encapsulating agent ratio; **S:EA:** Sample-encapsulating agent

**Table 3:** Responses of spray drying process in Inulin particles.

Run	Total Polar Content			Iridoids			Flavonoids			Phenylprop			Yield
	TC	EE	R	TC	EE	R	TC	EE	R	TC	EE	R	
1	133,745 ± 1561	77.27	49.97	3626 ± 38	80.07	76.05	6719 ± 225	83.74	53.62	123,400 ± 1298	75.88	47.63	70.07
2		73.78	43.51		76.14	54.67		84.54	39.62		72.12	43.31	69.83
3		77.44	51.99		78.55	59.98		84.18	48.24		76.43	51.99	73.53
4		74.40	44.52		76.86	50.48		86.81	37.48		72.69	45.15	68.56
5		74.90	46.77		74.86	57.90		82.88	40.62		73.93	46.91	71.18
6		67.01	16.82		67.53	18.96		84.17	12.03		65.22	17.38	28.51
7		75.22	51.05		75.81	56.70		86.38	39.59		73.93	52.36	74.47
8		75.85	49.17		74.07	51.26		84.33	38.46		75.02	50.61	74.70
9		67.29	27.12		66.61	27.88		83.67	18.24		65.78	28.38	53.79
10		76.83	47.03		78.71	43.13		87.77	34.30		75.59	49.18	65.43
11		72.56	31.67		73.15	33.20		85.19	22.88		71.22	32.86	58.88
12		71.40	47.75		73.59	46.45		85.78	32.99		69.85	50.02	71.27

**TC:** Total compounds content (Value = X ± SD. µg of analyte/g of microparticle.); **EE:** Encapsulation Efficiency; **R**: Recovery.

**Table 4:** Analysis of variance (ANOVA) of the regression models. Response variables: Yield, encapsulation and recovery of total polar content and both chemical group in inulin microparticles.

Source		Encapsulation				Recovery			
	Yield	Total Polar Content	Iridoids	Flavonoids	Phenylprop	Total Polar Content	Iridoids	Flavonoids	Phenylprop
	*p*-Value	*p*-Value	*p*-Value	*p*-Value	*p*-Value	*p*-Value	*p*-Value	*p*-Value	*p*-Value
**Model**	0.035 ^b^	0.000 ^b^	0.000 ^b^	0.457	0.000 ^b^	0.004 ^b^	0.003 ^b^	0.000 ^b^	0.010 ^b^
**X_1_:Temperature**	0.008 ^b^	0.006 ^b^	0.117	0.430	0.012 ^b^	0.092	0.023 ^b^	0.003 ^b^	0.162
**X_2_: S:EA ratio**	0.002 ^b^	0.000 ^b^	0.001 ^b^	0.613	0.001 ^b^	0.003 ^b^	0.001 ^b^	0.000 ^b^	0.005 ^b^
**X_1_ X_2_**	0.022 ^b^	0.029 ^b^	0.070	0.292	0.066	0.236	0.715	0.327	0.203
$X_{1}^{2}$	0.143	0.530	0.280	0.664	0.853	0.647	0.014 ^b^	0.006 ^b^	0.913
$X_{2}^{2}$	0.004 ^b^	0.034 ^b^	0.143	0.604	0.074	0.018 ^b^	0.032 ^b^	0.006 ^b^	0.023 ^b^
**Lack-of-fit**	0.032 ^b^	0.324	0.203	0.843	0.586	0.219	0.055	0.028 ^b^	0.250
**R^2^**	0.83	0.98	0.93	0.47	0.97	0.91	0.88	0.95	0.88
**CV**	<20	<1	<1	<1	<1	<15	<13	<12	<15

**R^2^** = Quadratic correlation coefficient; **CV** = coefficient of variation (%). ^b^ Significant (*p <* 0.050). **X_1_:** temperature; **X_2_:** Sample-encapsulating agent ratio; **S:EA:** Sample-encapsulating agent.

## Supplementary Materials

The following are available online at https://www.mdpi.com/2304-8158/9/11/1547/s1, Table S1: Experimental conditions of CCD; Table S2 Chemical composition of *L. citriodora* extract; Table S3: Encapsulation percentage from MD particles. Concentrations are exposed as µg of analyte/g of µparticle Value = X ± SD; Table S4: Recovery of compounds from MD microparticles. Concentrations are exposed as µg of analyte/g of µparticle. Value = X ± SD Table S5: Encapsulation percentage from inulin particles. Concentrations are exposed as µg of analyte/g of µparticle value = X ± SD; Table S6: Recovery of compounds from MD microparticles. Concentrations are exposed as µg of analyte/g of µparticle. Value = X ± SD.

## References

[1] Kolida S., Tuohy K., Gibson G.R. (2002). Prebiotic effects of inulin and oligofructose. Br. J. Nutr..

[2] Zhang J., Sun Z., Sun P., Chen T., Chen F. (2014). Microalgal carotenoids: Beneficial effects and potential in human health. Food Funct..

[3] Arab-Tehrany E., Jacquot M., Gaiani C., Imran M., Desobry S., Linder M. (2012). Beneficial effects and oxidative stability of omega-3 long-chain polyunsaturated fatty acids. Trends Food Sci. Technol..

[4] González Cañete N., Durán Agüero S. (2014). Soya isoflavones and evidences on cardiovascular protection. Nutr. Hosp..

[5] Melo M.N.D.O., Oliveira A.P., Wiecikowski A.F., Carvalho R.S., Castro J.D.L., De Oliveira F.A.G., Pereira H.M.G., Da Veiga V.F., Capella M.M.A., Rocha L. (2018). Phenolic compounds from Viscum album tinctures enhanced antitumor activity in melanoma murine cancer cells. Saudi Pharm. J..

[6] Sánchez-Marzo N., Lozano-Sánchez J., Cádiz-Gurrea M.D.L.L., Herranz-López M., Micol V., Segura-Carretero A. (2019). Relationships between chemical structure and antioxidant activity of isolated phytocompounds from lemon verbena. Antioxidants.

[7] Cádiz-Gurrea M.D.L.L., Micol V., Joven J., Segura-Carretero A., Fernández-Arroyo S. (2018). Different behavior of polyphenols in energy metabolism of lipopolysaccharide-stimulated cells. Food Res. Int..

[8] Diez-Echave P., Vezza T., Rodríguez-Nogales A., Hidalgo-Garcia L., Garrido-Mesa J., Ruiz-Malagon A., Molina-Tijeras J.A., Romero M., Robles-Vera I., Leyva-Jiménez F.J. (2020). The Beneficial Effects of Lippia Citriodora Extract on Diet-Induced Obesity in Mice Are Associated with Modulation in the Gut Microbiota Composition. Mol. Nutr. Food Res..

[9] Cádiz-Gurrea M.D.L.L., Olivares-Vicente M., Herranz-López M., Arráez-Román D., Fernández-Arroyo S., Micol V., Segura-Carretero A. (2018). Bioassay-guided purification of Lippia citriodora polyphenols with AMPK modulatory activity. J. Funct. Foods.

[10] Olivares-Vicente M., Sánchez-Marzo N., Encinar J.A., Cádiz-Gurrea M.D.L.L., Lozano-Sánchez J., Segura-Carretero A., Arraez-Roman D., Riva C., Barrajón-Catalán E., Herranz-López M. (2019). The Potential Synergistic Modulation of AMPK by Lippia citriodora Compounds as a Target in Metabolic Disorders. Nutrients.

[11] Nedovic V., Kalusevic A., Manojlovic V., Levic S., Bugarski B. (2011). An overview of encapsulation technologies for food applications. Procedia Food Sci..

[12] Karakaya S. (2004). Bioavailability of Phenolic Compounds. Crit. Rev. Food Sci. Nutr..

[13] Desai K.G.H., Jin Park H. (2005). Recent Developments in Microencapsulation of Food Ingredients. Dry. Technol..

[14] Shao P., Xuan S., Wu W., Qu L. (2019). Encapsulation efficiency and controlled release of Ganoderma lucidum polysaccharide microcapsules by spray drying using different combinations of wall materials. Int. J. Biol. Macromol..

[15] Tan S., Ebrahimi A., Langrish T. (2019). Controlled release of caffeine from tablets of spray-dried casein gels. Food Hydrocoll..

[16] Fang Z., Bhandari B. (2010). Encapsulation of polyphenols-A review. Trends Food Sci. Technol..

[17] Gibbs B.F., Kermasha S., Alli I., Mulligan C.N. (1999). Encapsulation in the food industry: A review. Int. J. Food Sci. Nutr..

[18] Paseephol T., Small D.M., Sherkat F. (2008). Rheology and texture of set yogurt as affected by inulin adition. J. Texture Stud..

[19] Leyva-Jiménez F.J., Lozano-Sánchez J., Borrás-Linares I., Arráez-Román D., Segura-Carretero A. (2019). Manufacturing design to improve the attainment of functional ingredients from Aloysia citriodora leaves by advanced microwave technology. J. Ind. Eng. Chem..

[20] Leyva-Jiménez F.J., Lozano-Sánchez J., Borrás-Linares I., Arráez-Román D., Segura-Carretero A. (2018). Comparative study of conventional and pressurized liquid extraction for recovering bioactive compounds from Lippia citriodora leaves. Food Res. Int..

[21] Oehlert G.W. (2010). A First Course in Design and Analysis of Experiments.

[22] Yusoff N.I., Leo C.P. (2017). Microwave Assisted Extraction of Defatted Roselle (*Hibiscus sabdariffa* L.) Seed at Subcritical Conditions with Statistical Analysis. J. Food Qual..

[23] Liyana-Pathirana C., Shahidi F. (2005). Optimization of extraction of phenolic compounds from wheat using response surface methodology. Food Chem..

[24] Fazaeli M., Emam-Djomeh Z., Kalbasi Ashtari A., Omid M. (2012). Effect of spray drying conditions and feed composition on the physical properties of black mulberry juice powder. Food Bioprod. Process..

[25] Gharsallaoui A., Roudaut G., Chambin O., Voilley A., Saurel R. (2007). Applications of spray-drying in microencapsulation of food ingredients: An overview. Food Res. Int..

[26] Tonon R.V., Brabet C., Hubinger M.D. (2010). Anthocyanin stability and antioxidant activity of spray-dried açai (*Euterpe oleracea* Mart.) juice produced with different carrier agents. Food Res. Int..

[27] Kha T.C., Nguyen M.H., Roach P.D. (2010). Effects of spray drying conditions on the physicochemical and antioxidant properties of the Gac (*Momordica cochinchinensis*) fruit aril powder. J. Food Eng..

[28] Reineccius G.A. (1988). Spray-Drying of Food Flavors. ACS Symp. Ser..

[29] Bazaria B., Kumar P. (2016). Compositional Changes in Functional Attributes of Vacuum Concentrated Beetroot Juice. J. Food Process. Preserv..

[30] Cádiz-Gurrea M., Lozano-Sánchez J., Fernández-Ochoa Á., Segura-Carretero A. (2019). Enhancing the Yield of Bioactive Compounds from Sclerocarya birrea Bark by Green Extraction Approaches. Molecules.

[31] Cai Y.Z., Corke H. (2000). Production and Properties of Spray-dried Amaranthus Betacyanin Pigments. J. Food Sci..

[32] Phoungchandang S., Sertwasana A. (2010). Spray-drying of ginger juice and physicochemical properties of ginger powders. Sci. Asia.

[33] Baur E., Ruhrberg K., Woishnis W. (2016). Chemical Resistance of Engineering Thermoplastics.

[34] Avaltroni F., Bouquerand P., Normand V. (2004). Maltodextrin molecular weight distribution influence on the glass transition temperature and viscosity in aqueous solutions. Carbohydr. Polym..

[35] Araujo-Díaz S.B., Leyva-Porras C., Aguirre-Bañuelos P., Álvarez-Salas C., Saavedra-Leos Z. (2017). Evaluation of the physical properties and conservation of the antioxidants content, employing inulin and maltodextrin in the spray drying of blueberry juice. Carbohydr. Polym..

[36] Kasapis S. (2005). Glass Transition Phenomena in Dehydrated Model Systems and Foods: A Review. Dry. Technol..

[37] Krishnan S., Bhosale R., Singhal R. (2005). Microencapsulation of cardamom oleoresin: Evaluation of blends of gum arabic, maltodextrin and a modified starch as wall materials. Carbohydr. Polym..

[38] Saenz C., Tapia S., Chavez J., Robert P. (2009). Microencapsulation by spray drying of bioactive compounds from cactus pear (*Opuntia ficus-indica*). Food Chem..

