# Supplementary material for: Spray-Drying Microencapsulation of Bioactive Compounds from Lemon Verbena Green Extract
Source: Foods. 2020 Oct 26;9(11):1547. doi: 10.3390/foods9111547 (PMC7692807; doi:10.3390/foods9111547)
Supplement: Supplementary file 1 [file foods-09-01547-s001.pdf]

foods

Stabilization of antioxidant and anti-inflammatory phytochemicals from lemon verbena Green extract by spray-drying microencapsulation.

Francisco Javier Leyva-Jiménez[1], Jesús Lozano-Sánchez[1,2*], María de la Luz Cádiz-Gurrea[1,3], Álvaro Fernández-Ochoa[1,3*], David Arráez-Román[1,3+], Antonio Segura-Carretero[1,3+].

[1] Functional Food Research and Development Center, Health Science Technological Park, Avenida del Conocimiento s/n, E-18016 Granada, Spain [2]; FJLJ: jleyva@cidaf.es

[2] Department of Food Science and Nutrition , University of Granada, Campus of Cartuja, 18071 Granada, Spain; JLS: jesusls@ugr.es

[3] Department of Analytical Chemistry, Faculty of Sciences, University of Granada, Fuentenueva s/n, E- 18071 Granada, Spain; MdlLCG: mluzcadiz@ugr.es; AFO: alvaroferochoa@ugr.es; DAR: darraez@ugr.es; ASC: ansegura@ugr.es.

[+] These authors are joint senior authors on this work

* Correspondence: jesusls@ugr.es; Tel.: +34-958-637083

Received: 20 September 2020; Accepted: 22 October 2020; Published: 26 October 2020

Table S1: Experimental conditions of CCD

Condition	Air Inlet Temperature (ºC)	S:EA ratio
**1**	165	13.84
**2**	165	9
**3**	140	13
**4**	165	9
**5**	165	9
**6**	190	5
**7**	165	9
**8**	134.75	9
**9**	165	4.16
**10**	190	13
**11**	140	5
**12**	195.25	9

Table S2: Chemical composition of *L. citriodora* extract.

Peak	RT (min)	*m/z* Cal	Formula (M-H)	Proposed compound	Quantification µg analyte/g extract
				Organic acids	
**1**	2.8	195.0510	$C_6H_{11}O_7$	Gluconic acid	NQ
	Iridoids glycosides				
**2**	3.8	373.1140	$C_{16}H_{21}O_{10}$	Gardoside	2662±22
**3**	3.9	391.1246	$C_{16}H_{23}O_{11}$	Shanzhiside	202±13
**4**	4.0	387.0933	$C_{16}H_{19}O_{11}$	Ixoside	1030±1
**10**	5.9	389.1089	$C_{16}H_{21}O_{11}$	Theveside	2720±4
**11**	7.4	449.1301	$C_{18}H_{25}O_{13}$	Myxopyroside	166±7
**12**	8.5	489.1614	$C_{21}H_{29}O_{13}$	Teucardoside	89±8
**20**	11.6	553.1563	$C_{25}H_{29}O_{14}$	Lippioside II	85±4
**24**	14.6	535.1457	$C_{25}H_{27}O_{13}$	Lippioside I derivarive	36±4
**25**	15.1	537.1614	$C_{25}H_{29}O_{13}$	Lippioside I	73±2
**33**	19.4	549.1614	$C_{26}H_{29}O_{13}$	Lippianoside B	8.9±0.5
**34**	19.8	521.1664	$C_{25}H_{29}O_{12}$	Hydroxycampsiside	45.868±0.005
**36**	20.7	551.1770	$C_{26}H_{31}O_{13}$	Durantoside I	126±22
**45**	31.4	569.2240	$C_{27}H_{37}O_{13}$	Manuleoside H	NQ
				Total iridoid glycosides	***7118 ± 87***
				Flavonoids	
**18**	10.6	637.1046	$C_{27}H_{25}O_{18}$	Luteolin-7-diglucoronide	2529±160
**23**	14.3	621.1097	$C_{27}H_{25}O_{17}$	Apigenin-7-diglucoronide	346±1
**27**	16.1	651.1355	$C_{28}H_{27}O_{18}$	Chrysoeriol-7-diglucuronide	5632±104
**38**	24.2	635.1254	$C_{28}H_{27}O_{17}$	Acacetin-7-diglucoronide	2093±58
**46**	32.1	315.0510	$C_{16}H_{11}O_7$	Methyl quercetin	27±3
**48**	34.7	299.0561	$C_{16}H_{11}O_6$	Dimethyl kaemferol	580±55
**49**	35.2	329.0667	$C_{17}H_{13}O_7$	Dimethyl quercetin	2242±74
				Total Flavonoids	***13449 ± 455***
				Phenylpropanoids	
**5**	4.2	461.1664	$C_{20}H_{29}O_{12}$	Verbasoside	3239±5
**6**	4.8	487.1457	$C_{21}H_{27}O_{13}$	Cistanoside F	1737±29

**15**	9.5	641.2087	$C_{29}H_{37}O_{16}$	β-Hydroxyverbascoside derivative	248±21
**16**	10.0	641.2087	$C_{29}H_{37}O_{16}$	β-Hydroxyisoverbascoside derivative	451±11
**17**	10.6	639.1931	$C_{29}H_{35}O_{16}$	β-Hydroxyverbascoside	1357±36
**19**	11.0	639.1931	$C_{29}H_{35}O_{16}$	β-Hydroxyisoverbascoside	1321±26
**22**	13.8	637.1774	$C_{29}H_{33}O_{16}$	Oxoverbascoside	42±2
**26**	15.6	653.2087	$C_{30}H_{37}O_{16}$	Campneoside I	0.58±0.01
**28**	16.4	623.1981	$C_{29}H_{35}O_{15}$	Verbascoside	173472±1483
**29**	17.9	521.2028	$C_{26}H_{33}O_{11}$	Lariciresinol glucopyranoside	1142±47
**30**	18.7	667.2244	$C_{31}H_{39}O_{16}$	Verbascoside A	864±16
**31**	18.9	623.1981	$C_{29}H_{35}O_{15}$	Isoverbascoside	54316±655
**32**	19.4	623.1981	$C_{29}H_{35}O_{15}$	Forsythoside A	692±33
**35**	20.2	607.2032	$C_{29}H_{35}O_{14}$	Lipedoside A I	259±11
**37**	21.3	637.2138	$C_{30}H_{37}O_{15}$	Leucoseptoside A	3516±73
**42**	27.2	651.2294	$C_{31}H_{39}O_{15}$	Martynoside or isomer	2349±104
**43**	29.6	651.2294	$C_{31}H_{39}O_{15}$	Martynoside or isomer	721±20
**44**	30.9	591.2083	$C_{29}H_{35}O_{13}$	Osmanthuside B	1090±31
				Total Phenylpropanoids	***246817 ± 2603***
			Others		
**8**	5.3	475.1398	$C_{20}H_{27}O_{13}$	Primeverin	NQ
**9**	5.8	285.0616	$C_{12}H_{13}O_{8}$	Pyrocatechol Glucuronide	NQ
**13**	8.7	387.1661	$C_{18}H_{27}O_{9}$	Tuberonic acid glucoside	NQ
			Unknown		
**7**	5.2	203.0925	$C_{9}H_{15}O_{5}$	UK 1	
**14**	9.0	433.2079	$C_{20}H_{33}O_{10}$	UK 2	
**21**	13.1	639.1872	$C_{36}H_{31}O_{11}$	UK 3	
**39**	25.2	551.2498	$C_{28}H_{39}O_{11}$	UK 4	
**40**	26.0	467.2134	$C_{20}H_{35}O_{12}$	UK 5	
**41**	26.3	549.1614	$C_{26}H_{29}O_{13}$	UK 6	
**47**	33.8	327.2177	$C_{18}H_{31}O_{5}$	UK 7	

***NQ:** Compound detected but its concentration is between detection and quantitation limits.

foods

MDPI

Table S3A: Encapsulation percentage from MD particles. Concentrations are exposed as μg of analyte/g of μparticle Value = X ± SD.

Compound		1	2	3	4	5	6
Shanzhiside	**Encapsulated**	28 ± 1	36.1 ± 0.3	33 ± 1	35 ± 1	37 ± 1	45 ± 2
	%EE	96.57	94.92	96.75	95.11	95.04	92.03
Gardoside	**Encapsulated**	143 ± 5	154 ± 12	149 ± 8	157 ± 8	164 ± 6	179 ± 10
	%EE	94.28	91.38	94.77	91.67	92.24	86.76
Ixoside	**Encapsulated**	83 ± 2	112 ± 3	101 ± 2	114 ± 2	117 ± 5	149 ± 5
	%EE	98.11	97.55	98.24	97.57	97.42	96.82
Theveside	**Encapsulated**	162 ± 4	216 ± 9	185 ± 5	235 ± 8	249 ± 7	321 ± 10
	%EE	98.28	97.93	98.57	98.11	98.13	97.21
Myxopyroside	**Encapsulated**	11.4 ± 0.4	24.7 ± 0.5	17.3 ± 0.3	25.3 ± 0.1	28.4 ± 0.1	46 ± 2
	%EE	93.43	92.82	95.10	93.02	93.51	89.85
Teucardoside	**Encapsulated**	1.89 ± 0.03	10.8 ± 0.4	5.5 ± 0.1	11.0 ± 0.1	13.7 ± 0.4	26.6 ± 0.6
	%EE	93.88	92.86	95.47	93.38	94.54	90.89
Lippioside II	**Encapsulated**	4.5 ± 0.1	16.7 ± 0.3	5.8 ± 0.1	18.6 ± 0.4	18.9 ± 0.7	36 ± 1
	%EE	96.94	95.79	96.71	96.53	96.51	94.58
Lippioside I Derivative	**Encapsulated**	0.99 ± 0.02	11.0 ± 0.2	3.2 ± 0.1	13.9 ± 0.2	13.5 ± 0.3	32.5 ± 0.8
	%EE	95.41	94.24	95.77	95.44	95.15	93.40
Lippioside I	**Encapsulated**	6.3 ± 0.1	17.5 ± 0.2	9.9 ± 0.1	20.010 ± 0.001	22.0 ± 0.5	41.1 ± 0.6
	%EE	93.23	92.84	94.60	93.16	93.40	89.55
Hydroxycampsiside	**Encapsulated**	4.9 ± 0.2	15.8 ± 0.3	7.6 ± 0.1	16.9 ± 0.1	17.84 ± 0.04	36 ± 2
	%EE	93.23	92.84	94.60	93.16	93.40	89.55
Lippianoside B	**Encapsulated**	1.24 ± 0.02	9.47 ± 0.09	2.95 ± 0.05	9.78 ± 0.07	12.1 ± 0.2	26.34 ± 0.07
	%EE	93.32	92.89	94.70	93.01	92.98	89.72
Durantoside I	**Encapsulated**	11.2 ± 0.1	21.6 ± 0.3	13.6 ± 0.3	24.3 ± 0.4	26.5 ± 0.5	45 ± 2
	%EE	90.89	91.67	93.35	92.15	92.31	88.17
Manuleoside H	**Encapsulated**	NQ	NQ	NQ	NQ	NQ	NQ
	%EE	50.38	55.07	55.77	56.91	56.16	52.46
Total iridoids	**Encapsulated**	**458 ± 13**	**646 ± 27**	**534 ± 17**	**681 ± 20**	**720 ± 22**	**984 ± 36**
	%EE	**94.82**	**93.86**	**95.68**	**94.25**	**94.41**	**91.65**

Compound		1	2	3	4	5	6
Luteolin-7 diglucuronide	**Encapsulated**	142.42 ± 0.01	188 ± 1	151 ± 2	199 ± 10	204 ± 4	262 ± 5
	**%EE**	99.75	99.79	99.69	99.73	99.76	99.79
Apigenin-7-diglucuronide	**Encapsulated**	20.0 ± 0.4	36 ± 1	22.6 ± 0.2	35.6 ± 0.1	40 ± 2	57 ± 1
	**%EE**	100.00	100.00	100.00	100.00	100.00	100.00
Chrysoeriol-7-diglucuronide	**Encapsulated**	260 ± 3	318.74 ± 0.03	281 ± 1	335 ± 7	342 ± 2	385 ± 2
	**%EE**	99.24	99.28	99.28	99.28	99.23	99.27
Acacenin-7-diglucuronide	**Encapsulated**	107 ± 1	148 ± 1	115 ± 1	151.4 ± 0.3	157 ± 2	191 ± 2
	**%EE**	99.12	99.34	99.28	99.30	99.33	99.37
Methylquercetin	**Encapsulated**	NQ	2.57 ± 0.05	NQ	3.68 ± 0.07	4.1 ± 0.1	10.8 ± 0.3
	**%EE**	72.04	72.48	75.36	75.70	75.04	68.79
Dimethyl kaempferol	**Encapsulated**	21 ± 1	30.7 ± 0.4	19 ± 1	28 ± 2	30 ± 1	42 ± 2
	**%EE**	64.93	63.26	67.86	65.86	68.14	61.55
Dimethyl quercetin	**Encapsulated**	85 ± 4	108 ± 4	82 ± 3	98 ± 2	109 ± 2	151 ± 7
	**%EE**	61.89	62.14	64.64	63.05	65.88	64.45
Total flavonoids	**Encapsulated**	**530 ± 4**	**687 ± 3**	**569 ± 5**	**721 ± 17**	**739 ± 10**	**895 ± 11**
	**%EE**	**86.77**	**86.83**	**88.80**	**88.44**	**89.07**	**86.98**
Verbasoside	**Encapsulated**	296 ± 6	316 ± 12	70 ± 2	384 ± 9	395 ± 16	437 ± 4
	**%EE**	94.63	92.38	81.93	93.79	93.68	88.45
Cistanoside F	**Encapsulated**	151 ± 4	203 ± 6	165 ± 5	219 ± 10	234 ± 5	306 ± 11
	**%EE**	96.71	95.87	96.61	96.29	96.27	93.66
β-Hydroxyverbascoside derivative	**Encapsulated**	17.2 ± 0.3	26.0 ± 0.2	18.5 ± 0.1	29 ± 1	31 ± 1	46 ± 1
	**%EE**	94.99	94.91	96.95	95.35	94.74	93.53
β-Hydroxyisoverbascoside derivative	**Encapsulated**	30 ± 1	45 ± 2	33 ± 1	50.2 ± 0.2	51 ± 1	77 ± 1
	**%EE**	96.77	95.38	97.35	96.60	96.83	95.13
β-Hydroxyverbascoside	**Encapsulated**	82 ± 2	127 ± 3	89 ± 1	131 ± 5	135 ± 6	191 ± 6
	**%EE**	95.75	95.19	95.85	95.31	96.07	92.48
β-Hydroxyisoverbascoside	**Encapsulated**	79 ± 2	115 ± 2	83 ± 5	119 ± 2	125 ± 2	163 ± 4
	**%EE**	95.34	95.32	96.00	95.53	95.59	92.15
Oxoverbascoside	**Encapsulated**	3.1 ± 0.1	9.2 ± 0.2	3.8 ± 0.2	8.7 ± 0.4	9.7 ± 0.4	21.3 ± 0.1
	**%EE**	95.12	94.22	94.69	94.72	94.49	93.70

Compound		**1**	**2**	**3**	**4**	**5**	**6**
Campneoside I	**Encapsulated**	NQ	1.8 ± 0.1	0.42 ± 0.02	3.1 ± 0.1	3.68 ± 0.03	7.9 ± 0.4
	**%EE**	91.41	90.96	91.72	93.27	95.14	90.80
Verbascoside	**Encapsulated**	1188 ± 8	1470 ± 14	1161 ± 2	1455 ± 20	1478 ± 8	1858 ± 34
	**%EE**	93.17	87.18	93.67	87.13	86.76	80.02
Lariciresinol glucopyranoside	**Encapsulated**	58.08 ± 0.04	81 ± 2	60 ± 2	87 ± 3	89 ± 5	123 ± 4
	**%EE**	87.23	86.74	89.23	87.69	87.53	80.72
Verbascoside A	**Encapsulated**	35 ± 1	68 ± 1	51 ± 1	68 ± 1	72.5 ± 0.3	99 ± 4
	**%EE**	87.82	86.84	92.51	89.43	88.43	84.07
Isoverbascoside	**Encapsulated**	347 ± 2	499 ± 15	348 ± 4	540 ± 5	548 ± 21	840 ± 9
	**%EE**	92.19	91.14	92.84	91.80	92.18	81.87
Forsythoside A	**Encapsulated**	173 ± 6	137 ± 8	195 ± 1	230 ± 2	241 ± 1	280 ± 3
	**%EE**	86.20	86.18	91.57	89.44	86.79	87.88
Lipedoside A I	**Encapsulated**	15.0 ± 0.5	24 ± 1	16.7 ± 0.5	26.4 ± 0.3	27 ± 1	41.0 ± 0.1
	**%EE**	90.32	90.33	91.78	90.77	91.19	86.27
Leucoseptoside A	**Encapsulated**	186 ± 8	243 ± 6	194 ± 1	267.7 ± 0.3	270 ± 5	349 ± 7
	**%EE**	85.80	85.82	87.84	87.26	87.20	80.43
Martynoside	**Encapsulated**	120 ± 6	174 ± 5	135 ± 2	177 ± 4	182 ± 7	238 ± 5
	**%EE**	73.43	77.02	79.24	79.27	79.03	73.22
Martynoside isomer	**Encapsulated**	35 ± 1	55 ± 1	39 ± 1	56.35 ± 0.03	59 ± 2	85 ± 2
	**%EE**	69.06	73.51	74.90	75.76	76.00	71.09
Osmanthisude B	**Encapsulated**	56 ± 2	83 ± 2	63 ± 1	88 ± 2	92 ± 2	126.8 ± 0.4
	**%EE**	68.10	71.48	72.78	73.53	73.84	68.25
Total phenylpropanoids	**Encapsulated**	**2870 ± 48**	**3675 ± 81**	**2726 ± 31**	**3935 ± 66**	**4039 ± 83**	**5282 ± 95**
	**%EE**	**92.20**	**88.29**	**92.83**	**88.69**	**88.52**	**81.18**
Total polar compounds	**Encapsulated**	**3858 ± 65**	**5008 ± 111**	**3829 ± 53**	**4717 ± 103**	**5498 ± 115**	**7161 ± 142**
	**%EE**	**91.69**	**88.47**	**92.51**	**89.04**	**89.00**	**82.56**

*NQ: Compound detected but its concentration is between detection and quantitation limits.

Table S3B: *Encapsulation percentage from MD particles. Concentrations are exposed as µg of analyte/g of µparticle Value = X ± SD.*

**Compound**		**7**	**8**	**9**	**10**	**11**	**12**
Shanzhiside	**Encapsulated**	37 ± 1	35.7 ± 0.4	48 ± 2	27.87 ± 0.02	42 ± 1	42 ± 1
	**%EE**	95.04	95.20	90.66	95.70	90.61	94.55
Gardoside	**Encapsulated**	167 ± 4	161 ± 10	183 ± 7	141 ± 5	177 ± 3	172 ± 6
	**%EE**	92.01	92.12	83.59	93.42	84.97	90.74
Ixoside	**Encapsulated**	112 ± 4	116 ± 5	158 ± 5	96 ± 3	137 ± 3	132 ± 2
	**%EE**	97.37	97.89	96.47	98.00	96.38	97.49
Theveside	**Encapsulated**	221 ± 6	241 ± 13	369 ± 10	186 ± 2	318 ± 14	256 ± 7
	**%EE**	98.02	98.12	96.84	98.23	96.69	97.84
Myxopyroside	**Encapsulated**	27 ± 1	27.2 ± 0.3	53 ± 3	14.4 ± 0.1	40 ± 1	28 ± 1
	**%EE**	93.86	93.66	88.30	93.09	87.76	90.97
Teucardoside	**Encapsulated**	12.5 ± 0.3	13.1 ± 0.1	34.1 ± 0.8	4.3 ± 0.1	23.8 ± 0.4	12.5 ± 0.3
	**%EE**	94.34	93.76	89.13	94.38	88.31	91.24
Lippioside II	**Encapsulated**	16.9 ± 0.6	20.3 ± 0.3	46 ± 1	9.6 ± 0.4	39.5 ± 0.9	18.2 ± 0.2
	**%EE**	96.79	96.55	93.55	96.58	93.81	95.55
Lippioside I Derivative	**Encapsulated**	12.9 ± 0.2	15.9 ± 0.6	40 ± 2	6.0 ± 0.2	31.2 ± 0.9	15.6 ± 0.1
	**%EE**	95.13	95.40	91.97	95.81	92.04	94.35
Lippioside I	**Encapsulated**	19.0 ± 0.5	20.9 ± 2.4	49.7 ± 0.9	11.3 ± 0.4	38.6 ± 1.6	21.8 ± 0.5
	**%EE**	93.54	93.38	87.58	93.61	88.51	91.70
Hydroxycampsiside	**Encapsulated**	17.8 ± 0.2	19.4 ± 0.3	41 ± 2	7.8 ± 0.1	34 ± 1	19.79 ± 0.04
	**%EE**	93.54	93.38	87.58	93.61	88.51	91.70
Lippianoside B	**Encapsulated**	10.7 ± 0.1	12.5 ± 0.2	32.5 ± 0.8	4.0 ± 0.1	23.8 ± 0.6	12.62 ± 0.08
	**%EE**	93.09	93.05	88.42	93.42	88.49	91.68
Durantoside I	**Encapsulated**	26.2 ± 0.1	27.1 ± 0.3	54 ± 2	14.8 ± 0.2	45 ± 2	27 ± 1
	**%EE**	91.92	92.32	87.07	92.26	87.29	91.05
Manuleoside H	**Encapsulated**	NQ	NQ	NQ	NQ	NQ	NQ
	**%EE**	63.68	65.59	23.55	76.60	24.96	47.98
Total iridoids	**Encapsulated**	**680 ± 19**	**710 ± 33**	**1109 ± 38**	**524 ± 10**	**950 ± 29**	**758 ± 18**
	**%EE**	**94.27**	**94.44**	**90.49**	**94.77**	**90.55**	**93.31**

**Compound**		**7**	**8**	**9**	**10**	**11**	**12**
Luteolin-7 diglucuronide	**Encapsulated**	201 ± 1	210 ± 3	284 ± 4	162 ± 2	259 ± 4	200 ± 2
	**%EE**	99.73	99.73	99.78	99.75	99.75	99.74
Apigenin-7-diglucuronide	**Encapsulated**	35 ± 1	42 ± 1	75 ± 1	26.8 ± 0.4	61 ± 2	39.0 ± 0.4
	**%EE**	100.00	100.00	100.00	100.00	100.00	100.00
Chrysoeriol-7-diglucuronide	**Encapsulated**	318 ± 1	327 ± 4	402 ± 2	286 ± 1	359 ± 1	304 ± 1
	**%EE**	99.32	99.18	99.21	99.21	99.23	99.15
Acacenin-7-diglucuronide	**Encapsulated**	150 ± 4	155 ± 6	210 ± 4	119 ± 4	193 ± 2	152 ± 2
	**%EE**	99.32	99.24	99.46	99.22	99.42	99.27
Methylquercetin	**Encapsulated**	2.17 ± 0.03	5.4 ± 0.1	13.1 ± 0.5	1.97 ± 0.06	11.3 ± 0.5	6.26 ± 0.9
	**%EE**	72.93	74.13	65.78	74.16	66.38	73.80
Dimethyl kaempferol	**Encapsulated**	26 ± 1	29 ± 0.2	46 ± 1	17.9 ± 0.3	46 ± 1	28 ± 2
	**%EE**	65.84	66.89	64.71	66.89	63.75	65.46
Dimethyl quercetin	**Encapsulated**	100 ± 5	114 ± 7	158 ± 1	76 ± 1	153 ± 1	98 ± 3
	**%EE**	63.85	63.62	63.78	63.71	63.04	62.64
Total Flavonoids	**Encapsulated**	**701 ± 8**	**732 ± 15**	**965 ± 11**	**591 ± 7**	**872 ± 8**	**694 ± 6**
	**%EE**	**88.41**	**87.93**	**87.10**	**88.95**	**86.21**	**87.79**
Verbasoside	**Encapsulated**	354 ± 8	389 ± 9	490 ± 10	329 ± 6	453 ± 8	388 ± 7
	**%EE**	93.42	93.24	86.94	94.07	87.32	91.95
Cistanoside F	**Encapsulated**	207 ± 2	232 ± 7	354 ± 3	170 ± 1	299 ± 13	226 ± 5
	**%EE**	96.04	95.58	92.00	96.11	91.51	94.67
β-Hydroxyverbascoside derivative	**Encapsulated**	28.2 ± 0.2	31 ± 1	52 ± 2	20 ± 1	44 ± 1	28 ± 1
	**%EE**	95.68	94.97	91.77	95.95	92.58	94.48
β-Hydroxyisoverbascoside derivative	**Encapsulated**	47.8 ± 0.4	52 ± 1	86 ± 2	37 ± 1	75 ± 1	49 ± 1
	**%EE**	96.81	96.05	93.22	97.36	92.90	94.35
β-Hydroxyverbascoside	**Encapsulated**	125 ± 4	137 ± 2	218 ± 7	100 ± 2	195 ± 1	129 ± 4
	**%EE**	95.65	94.53	90.56	95.56	91.37	93.62
β-Hydroxyisoverbascoside	**Encapsulated**	111 ± 5	118 ± 4	178 ± 4	92 ± 1	161 ± 4	116 ± 1
	**%EE**	95.10	95.27	89.72	95.84	90.38	93.67
Oxoverbascoside	**Encapsulated**	9.42 ± 0.04	10.1 ± 0.4	24.2 ± 0.3	6.4 ± 0.3	19.9 ± 1.1	10.5 ± 0.3
	**%EE**	93.70	94.21	90.73	94.50	91.64	92.43

Compound		7	8	9	10	11	12
Campneoside I	**Encapsulated**	3.1 ± 0.1	4.0 ± 0.1	9.9 ± 0.4	1.41 ± 0.03	7.6 ± 0.3	3.5 ± 0.1
	**%EE**	93.97	93.50	90.29	92.22	88.53	88.12
Verbascoside	**Encapsulated**	1371 ± 29	1407 ± 35	1985 ± 44	1103 ± 31	1767 ± 12	1317 ± 6
	**%EE**	85.46	92.31	77.38	92.43	77.99	84.59
Lariciresinol glucopyranoside	**Encapsulated**	81 ± 1	85 ± 1	143 ± 6	59 ± 1	124 ± 3	78 ± 2
	**%EE**	87.27	86.50	80.43	84.99	80.47	81.90
Verbascoside A	**Encapsulated**	68 ± 1	72 ± 1	105 ± 5	53 ± 1	93 ± 1	69.4 ± 0.3
	**%EE**	88.48	88.13	83.22	89.93	83.99	83.56
Isoverbascoside	**Encapsulated**	503 ± 5	488 ± 6	907 ± 8	333 ± 4	762 ± 6	455 ± 10
	**%EE**	91.59	91.20	79.65	91.21	79.41	88.98
Forsythoside A	**Encapsulated**	210 ± 2	215 ± 1	214 ± 6	181 ± 5	250 ± 5	213 ± 3
	**%EE**	90.20	87.60	76.53	89.54	84.39	87.15
Lipedoside A I	**Encapsulated**	25.3 ± 0.3	27.9 ± 0.2	47 ± 1	19.3 ± 0.2	40 ± 1	25.4 ± 0.2
	**%EE**	91.16	90.20	83.67	90.41	84.76	86.44
Leucoseptoside A	**Encapsulated**	258 ± 8	270 ± 12	365 ± 7	210 ± 6	348 ± 4	253 ± 6
	**%EE**	86.84	86.89	78.12	86.61	79.48	81.76
Martynoside	**Encapsulated**	181 ± 3	182 ± 5	264 ± 5	134 ± 1	247 ± 2	172 ± 2
	**%EE**	79.84	79.27	72.01	75.33	73.89	71.62
Martynoside isomer	**Encapsulated**	58 ± 1	58.9 ± 0.4	98 ± 2	41 ± 1	84 ± 2	54.4 ± 0.4
	**%EE**	76.41	75.71	71.60	71.41	71.44	68.60
Osmanthisude B	**Encapsulated**	83 ± 2	89 ± 6	137 ± 2	66 ± 1	119 ± 5	81 ± 2
	**%EE**	73.36	72.64	66.50	69.55	66.12	65.34
Total Phenylpropanoids	**Encapsulated**	**3722 ± 71**	**3862 ± 91**	**5667 ± 114**	**2954 ± 63**	**5079 ± 70**	**3665 ± 51**
	**%EE**	**87.61**	**91.45**	**78.70**	**91.51**	**79.13**	**85.85**
Total polar compounds	**Encapsulated**	**5103 ± 98**	**5304 ± 139**	**7741 ± 163**	**4069 ± 80**	**6901 ± 107**	**5117 ± 75**
	**%EE**	**88.17**	**91.19**	**80.47**	**91.41**	**80.75**	**86.67**

*NQ: Compound detected but its concentration is between detection and quantitation limits.

Table S4: Recovery of compounds from MD microparticles. Concentrations are exposed as μg of analyte/g of μparticle. Value = X ± SD.

Compound	Variable	1	2	3	4	5	6	7	8	9	10	11	12
Shanzhiside (Total fed: 101 ± 7)	**%R**	78.75	55.33	82.45	57.00	59.24	23.08	59.19	57.02	16.20	73.70	17.53	44.67
Gardoside (Total fed: 1330 ± 11)	**%R**	54.63	35.91	54.44	38.62	39.78	15.16	40.18	38.48	10.58	50.92	12.00	29.16
Ixoside (Total fed: 514.8 ± 0.5)	**%R**	70.90	54.01	79.96	58.11	59.27	24.37	56.73	57.83	17.07	74.59	17.82	45.50
Theveside (Total fed: 1360 ± 2)	**%R**	57.55	44.46	62.16	50.75	53.40	22.65	47.56	51.16	17.12	60.83	17.68	38.06
Myxopyroside (Total fed: 83 ± 4)	**%R**	57.40	48.15	64.03	51.47	54.51	25.34	52.86	52.33	18.73	58.54	18.51	38.64
Teucardoside (Total fed:44 ± 4)	**%R**	46.71	38.76	51.10	41.02	43.72	20.59	42.01	42.74	16.02	47.87	15.64	30.29
Lippioside II (Total fed: 43 ± 2)	**%R**	50.62	44.69	51.44	49.17	49.54	23.64	46.54	50.48	18.56	56.65	19.92	33.99
Lippioside I Derivative (Total fed: 18 ± 2)	**%R**	49.17	42.68	51.95	48.58	48.03	24.96	46.92	50.44	19.15	55.80	19.39	35.53
Lippioside I (Total fed: 36.2 ± 0.8)	**%R**	55.91	47.41	60.69	52.64	55.39	27.03	51.09	52.89	20.51	61.90	20.62	38.56
Hydroxycampsiside (Total fed: 22.93 ± 0.02)	**%R**	57.73	48.66	60.46	52.85	53.87	27.16	53.28	55.11	19.85	59.52	20.64	39.82
Lippianoside B (Total fed: 4.5 ± 0.3)	**%R**	53.92	43.81	55.13	46.75	50.09	24.47	47.67	49.97	18.48	56.24	18.40	35.70
Durantoside I (Total fed: 69 ± 4)	**%R**	61.34	47.74	61.97	53.35	55.72	26.90	55.15	55.62	20.43	62.63	21.21	39.72
Manuleoside H (Total fed: NQ)	**%R**	75.81	59.86	71.19	66.13	66.01	32.51	63.68	65.59	23.55	76.60	24.96	47.98
**Total iridoids (Total fed: 3626 ± 38)**	**%R**	**58.71**	**44.59**	**61.95**	**48.85**	**50.66**	**21.99**	**48.36**	**49.43**	**16.19**	**59.84**	**16.96**	**36.79**

Compound	Variable	1	2	3	4	5	6	7	8	9	10	11	12
Luteolin-7 diglucuronide (Total fed: 1260 ± 80)	%R	54.20	41.73	55.22	46.63	47.61	20.01	46.62	48.27	14.28	56.98	15.48	32.30
Apigenin-7-diglucuronide (Total fed: 173.2 ± 0.3)	%R	52.85	46.30	54.97	48.87	52.74	23.27	47.79	54.16	19.23	59.29	19.23	36.10
Chrysoeriol-7-diglucuronide (Total fed: 2816 ± 51)	%R	45.30	32.79	47.07	36.37	37.04	13.78	34.30	35.05	9.50	46.36	10.09	22.93
Acacenin-7-diglucuronide (Total fed: 1046 ± 28)	%R	50.03	39.89	51.36	43.03	44.50	17.68	42.17	43.21	12.76	51.09	13.97	29.81
Methyl quercetin (Total fed: 14 ± 2)	%R	50.96	43.02	53.35	46.32	47.70	23.43	43.78	50.89	17.71	63.56	19.38	37.45
Dimethyl kaempferol (Total fed: 289 ± 27)	%R	61.03	47.65	53.32	44.89	46.08	21.17	43.31	44.84	14.50	50.65	17.27	31.96
Dimethyl quercetin (Total fed: 1121 ± 37)	%R	61.36	45.33	55.16	43.17	45.38	20.48	43.05	48.18	14.39	50.85	16.63	30.14
**Total flavonoids (Total fed: 6719 ± 225)**	%R	**52.30**	**39.69**	**51.60**	**41.74**	**43.07**	**17.87**	**40.65**	**42.92**	**12.69**	**50.95**	**13.97**	**28.45**
Verbasoside (Total fed:1619 ± 3)	%R	91.87	59.73	26.03	75.45	77.56	30.09	69.38	75.54	22.61	95.65	24.72	53.71
Cistanoside F (Total fed: 868 ± 14)	%R	84.49	67.30	88.93	76.29	81.33	36.00	71.91	79.95	27.96	89.01	28.23	55.35
β-Hydroxyverbascoside derivative (Total fed: 124 ± 10)	%R	63.50	51.61	63.45	58.85	62.29	29.63	57.21	61.23	22.10	66.04	22.30	40.03
β-Hydroxyisoverbascoside derivative (Total fed: 225 ± 5)	%R	63.54	53.73	65.38	61.17	61.52	30.12	57.94	62.86	22.59	68.92	23.72	42.41
β-Hydroxyverbascoside (Total fed: 678 ± 17)	%R	60.02	54.37	62.68	58.90	60.11	28.99	55.90	61.40	22.15	67.71	23.38	41.12
β-Hydroxyisoverbascoside (Total fed: 661 ± 13)	%R	59.67	50.57	60.34	55.02	57.68	25.61	51.36	54.12	18.82	64.23	20.11	37.96
Oxoverbascoside (Total fed: 20.8 ± 0.8)	%R	56.73	54.34	58.88	55.27	58.86	33.25	58.05	59.55	25.03	70.28	25.38	43.61

Compound	Variable	1	2	3	4	5	6	7	8	9	10	11	12
Campneoside I (Total fed: 0.29 ± 0.01)	%R	ND	39.77	53.95	46.97	48.45	23.44	46.13	49.91	17.57	58.61	18.43	35.45
Verbascoside (Total fed: 86735 ± 741)	%R	43.32	32.84	40.89	34.35	35.02	15.18	33.19	31.10	11.03	38.62	11.69	22.55
Lariciresinol glucopyranoside (Total fed: 570 ± 23)	%R	56.48	45.68	55.22	50.95	52.26	25.37	47.89	50.08	19.50	55.00	20.06	34.16
Verbascoside A (Total fed: 431 ± 8)	%R	46.29	49.58	58.53	50.95	54.69	25.48	51.33	53.48	17.89	59.90	18.71	38.48
Isoverbascoside (Total fed: 27158 ± 327)	%R	58.47	43.27	56.13	47.50	47.69	23.72	45.20	44.27	17.11	54.01	17.87	30.58
Forsythoside A (Total fed: 346 ± 16)	%R	54.28	41.57	56.86	46.60	48.42	20.27	43.99	42.42	15.27	54.31	15.81	28.87
Lipedoside A I (Total fed: 129 ± 6)	%R	59.35	48.98	60.65	56.36	56.66	28.16	53.76	58.23	21.50	65.88	21.94	39.57
Leucoseptoside A (Total fed: 1757 ± 37)	%R	59.69	46.10	58.64	52.67	53.10	24.54	50.57	52.43	17.45	62.00	19.41	36.71
Martynoside (Total fed: 1174 ± 52)	%R	67.31	54.70	67.31	57.13	58.78	27.26	57.38	57.53	20.20	68.15	21.87	42.44
Martynoside isomer (Total fed: 360 ± 10)	%R	67.80	56.94	66.47	59.38	61.93	30.54	60.20	60.83	22.76	70.84	23.46	43.92
Osmanthisude B (Total fed: 544 ± 15)	%R	73.27	59.35	73.75	64.24	66.47	32.26	60.46	64.78	23.53	77.65	24.56	46.04
**Total phenylpropanoids (Total fed: 123400 ± 1298)**	%R	**49.41**	**37.18**	**46.48**	**39.99**	**40.69**	**18.40**	**38.38**	**37.04**	**13.34**	**45.44**	**14.11**	**26.39**
**Total polar compounds (Total fed: 133745 ± 1561)**	%R	**50.31**	**37.90**	**47.98**	**40.70**	**41.55**	**18.54**	**39.22**	**38.45**	**13.42**	**46.93**	**14.26**	**27.23**

Table S5A: Encapsulation percentage from inulin particles. Concentrations are exposed as μg of analyte/g of μparticle value = X ± SD.

Compound		1	2	3	4	5	6
Shanzhiside	**Encapsulated**	34±1	25.2±0.9	22.8±0.4	22.8±0.6	30.6±0.5	36±4
	**%EE**	94.16	91.91	93.02	91.29	90.66	86.83
Gardoside	**Encapsulated**	151±4	124±7	105±6	119±5	128±4	129±12
	**%EE**	56.42	46.08	53.47	47.53	43.84	31.62
Ixoside	**Encapsulated**	96±4	86±7	63±5	84±8	90±3	112±11
	**%EE**	98.11	97.41	96.77	97.50	97.35	97.77
Theveside	**Encapsulated**	157±15	177±9	120±10	150±7	179±3	235±2
	**%EE**	97.02	96.90	96.44	96.74	96.54	96.25
Myxopyroside	**Encapsulated**	11±1	11.51±0.05	1.02±0.02	13.90±0.13	13±1	24±1
	**%EE**	87.59	83.74	84.37	85.81	82.92	76.07
Teucardoside	**Encapsulated**	2.42±0.03	2.9±0.1	NQ	2.7±0.0	1.7±0.1	22.6±0.4
	**%EE**	87.12	84.64	84.32	85.41	82.63	81.71
Lippioside II	**Encapsulated**	3.8±0.2	9.0±0.2	1.302±0.005	8.0±0.1	8.8±0.3	19.1±0.4
	**%EE**	91.92	90.78	92.14	90.53	89.44	84.43
Lippioside I Derivative	**Encapsulated**	2.34±0.01	5.4±0.1	NQ	5.1±0.3	4.58±0.01	16±1
	**%EE**	90.29	89.15	89.80	89.62±	88.00	84.59
Lippioside I	**Encapsulated**	6.8±0.1	11.1±0.2	1.62±0.03	10.2±0.2	11.4±0.1	22.5±1.0
	**%EE**	88.84	86.43	87.05	87.77	86.65	82.93
Hydroxycampsiside	**Encapsulated**	4.1±0.1	8.0±0.2	NQ	7.7±0.5	7.6±0.6	17.1±0.5
	**%EE**	84.43	83.18	83.95	84.62	81.51	77.27
Lippianoside B	**Encapsulated**	1.9±0.1	8.6±0.1	3.0±0.2	10.6±0.4	10.4±0.4	19.3±0.6
	**%EE**	88.86	84.05	86.89	86.78	82.99	73.43
Durantoside I	**Encapsulated**	7.3±0.3	16.7±0.1	5.18±0.08	15.7±0.6	15.7±0.9	25.5±0.2
	**%EE**	83.38	83.79	84.43	84.04	82.15	73.41
Manuleoside H	**Encapsulated**	NQ	NQ	NQ	NQ	NQ	NQ
	**%EE**	39.44	44.68	40.41	45.47	43.23	39.72
Total iridoids	**Encapsulated**	**478±26**	**486±25**	**322±22**	**449±24**	**500±13**	**679±34**
	**%EE**	**80.07**	**76.14**	**78.55**	**76.86**	**74.36**	**67.53**

Compound		**1**	**2**	**3**	**4**	**5**	**6**
Luteolin-7 diglucuronide	**Encapsulated**	143±3	164±3	132±4	161±5	161±7	216±9
	**%EE**	99.61	99.78	99.65	99.69	99.72	100.00
Apigenin-7-diglucuronide	**Encapsulated**	20.2±0.3	27±1	22±1	27±1	27±1	44±2
	**%EE**	100.00	100.00	100.00	100.00	100.00	100.00
Chrysoeriol-7-diglucuronide	**Encapsulated**	262±6	269±2	234±1	267±5	265±8	301±6
	**%EE**	98.95	99.05	98.88	99.03	99.00	99.30
Acacenin-7-diglucuronide	**Encapsulated**	101±2	121.2±0.2	97±3	120±4	120±6	157±4
	**%EE**	98.93	99.18	99.07	99.28	99.12	99.44
Methylquercetin	**Encapsulated**	NQ	NQ	NQ	NQ	NQ	1.74± 0.05
	**%EE**	51.48	55.18	53.70	58.20	53.68	54.81
Dimethyl kaempferol	**Encapsulated**	8.0±0.2	12.1±0.1	7.9±0.1	14.8±0.8	16.7±0.9	20.3±1.1
	**%EE**	48.88	50.94	52.79	58.85	52.02	52.47
Dimethyl quercetin	**Encapsulated**	59±1	71±2	45±2	67±3	64±3	95±4
	**%EE**	52.35	55.02	49.53	58.89	50.18	55.08
Total flavonoids	**Encapsulated**	**593±13**	**664±9**	**538±12**	**656±19**	**654±26**	**835±26**
	**%EE**	**83.74**	**84.54**	**84.18**	**86.81**	**82.88**	**84.17**
Verbasoside	**Encapsulated**	266±8	280±14	236±21	283±24	298±8	333±6
	**%EE**	88.03	85.82	87.93	85.82	86.11	78.12
Cistanoside F	**Encapsulated**	144±8	163±10	135±19	155±13	166±3	211±4
	**%EE**	90.03	88.31	90.60	89.41	89.18	81.97
β-Hydroxyverbascoside derivative	**Encapsulated**	15.3±0.2	19.2±0.3	14.5±0.5	19±1	19±1	27.8±0.2
	**%EE**	89.07	86.53	90.29	86.61	87.30	78.16
β-Hydroxyisoverbascoside derivative	**Encapsulated**	29±1	36.55±0.03	27.3±0.4	35.9±0.4	36±2	51±2
	**%EE**	89.84	90.26	92.04	89.73	88.30	81.87
β-Hydroxyverbascoside	**Encapsulated**	75±3	92±3	71±2	94±2	93±2	131±8
	**%EE**	86.41	85.40	87.72	87.27	84.80	76.11
β-Hydroxyisoverbascoside	**Encapsulated**	68±2	82±2	69±2	85±2	89±3	114±3
	**%EE**	86.68	84.98	89.01	86.89	85.13	76.74

Compound		1	2	3	4	5	6
Oxoverbascoside	**Encapsulated**	3.3±0.1	5.9±0.4	2.9±0.2	6.2±0.1	6.1±0.2	10.9±0.5
Oxoverbascoside	**%EE**	86.82	82.17	86.01	86.02	84.88	77.71
Campneoside I	**Encapsulated**	0.245±0.004	0.70±0.06	0.019±0.001	0.96±0.05	1.30±0.06	4.8±0.2
Campneoside I	**%EE**	85.00	81.92	88.46	82.36	83.43	82.69
Verbascoside	**Encapsulated**	797±11	1117±13	955±7	1235±16	1265±13	1809±5
Verbascoside	**%EE**	75.80	71.55	76.82	72.34	73.82	64.66
Lariciresinol glucopyranoside	**Encapsulated**	34.2±0.5	45±1	29.3±0.2	45±1	45±3	65±1
Lariciresinol glucopyranoside	**%EE**	68.33	67.27	66.96	69.77	66.25	59.91
Verbascoside A	**Encapsulated**	33±1	40±2	33±1	40.4±0.2	42±1	54±1
Verbascoside A	**%EE**	71.83	71.82	78.96	72.79	71.35	65.29
Isoverbascoside	**Encapsulated**	132.0±0.4	294±5	197±6	350±2	362±10	676±4
Isoverbascoside	**%EE**	76.22	72.07	76.23	72.71	74.07	65.49
Forsythoside A	**Encapsulated**	126±7	141±5	91±6	145±3	139±3	145±4
Forsythoside A	**%EE**	60.76	60.58	49.94	54.40	58.27	53.80
Lipedoside A I	**Encapsulated**	14.4±0.8	19.1±0.1	11.70±0.04	17.9±0.4	17.7±0.7	24±1
Lipedoside A I	**%EE**	75.03	74.53	69.99	72.64	71.75	63.85
Leucoseptoside A	**Encapsulated**	164±7	196±4	159±10	192±2	194±7	241±8
Leucoseptoside A	**%EE**	74.64	73.63	74.27	73.77	71.38	64.16
Martynoside	**Encapsulated**	97±1	126±3	98±7	126±9	122±3	165±5
Martynoside	**%EE**	61.97	65.13	63.86	66.62	63.59	58.48
Martynoside isomer	**Encapsulated**	28±1	39±1	27.681±0.003	36.6±0.3	36±1	56±1
Martynoside isomer	**%EE**	60.55	64.80	62.76	63.98	62.43	59.51
Osmanthisude B	**Encapsulated**	46±1	55±1	40±1	53±1	55±3	74±3
Osmanthisude B	**%EE**	54.20	56.85	53.14	56.89	54.04	51.22
Total phenylpropanoids	**Encapsulated**	**2072±53**	**2752±64**	**2198±83**	**2918±78**	**2985±65**	**4192±57**
Total phenylpropanoids	**%EE**	**75.88**	**72.12**	**76.43**	**72.69**	**73.93**	**65.22**
Total polar compounds	**Encapsulated**	3143±92	3902±98	3058±117	4023±121	4139±104	5706±117
Total polar compounds	**%EE**	77.27	73.78	77.44	74.40	74.90	67.01

*NQ: Compound detected but its concentration is between detection and quantitation limits.

Table S5B: Encapsulation percentage from inulin particles. Concentrations are exposed as µg of analyte/g of µparticle value = X ± SD.

Compound		**7**	**8**	**9**	**10**	**11**	**12**
Shanzhiside	**Encapsulated**	28±2	24.6±0.8	26.1±0.6	9.3±0.4	26.0±0.6	18.5±0.4
	**%EE**	91.74	90.85	85.53	92.24	88.44	90.73
Gardoside	**Encapsulated**	125±1	109±7	122±3	77±6	124±3	108±4
	**%EE**	46.19	44.56	31.94	51.66	38.93	45.35
Ixoside	**Encapsulated**	86±6	70±5	91±2	46±2	92±7	68±3
	**%EE**	97.56	97.03	97.29	97.88	97.40	97.15
Theveside	**Encapsulated**	164±3	143±10	212±10	95±6	224±4	131±5
	**%EE**	96.92	96.17	95.86	97.44	96.89	96.73
Myxopyroside	**Encapsulated**	10.98±0.08	6.64±0.46	17.45±0.43	NQ	21±1	4.74±0.23
	**%EE**	84.48	82.03	76.29	86.03	81.72	81.35
Teucardoside	**Encapsulated**	1.6±0.1	NQ	5.5±0.2	NQ	7.2±0.0	NQ
	**%EE**	84.78	82.32	74.19	85.93	80.71	81.46
Lippioside II	**Encapsulated**	7.3±0.2	3.8±0.1	18.0±0.2	NQ	17.5±0.4	2.4±0.1
	**%EE**	90.34	89.83	85.25	92.69	87.79	88.22
Lippioside I Derivative	**Encapsulated**	3.9±0.1	0.12±0.01	12.0±0.1	NQ	13.1±0.5	0.33±0.01
	**%EE**	87.81	87.62	83.67	90.32	86.30	87.12
Lippioside I	**Encapsulated**	9.8±0.2	5.4±0.2	18.9±0.4	NQ	18.2±0.3	3.30±0.01
	**%EE**	87.51	86.34	81.42	89.63	84.67	84.14
Hydroxycampsiside	**Encapsulated**	5.95±0.03	3.0±0.2	15.1±0.3	NQ	19.4±0.5	3.2±0.1
	**%EE**	83.38	81.76	75.16	86.42	81.76	80.56
Lippianoside B	**Encapsulated**	8.3±0.1	2.7±0.1	17.6±0.9	NQ	14.9±0.4	NQ
	**%EE**	85.46	80.00	75.52	89.36	84.14	81.12
Durantoside I	**Encapsulated**	12.8±0.3	8.7±0.3	23.0±0.2	1.54±0.02	24.4±0.3	7.84±0.02
	**%EE**	82.67	82.32	73.94	85.19	79.34	79.93
Manuleoside H	**Encapsulated**	NQ	NQ	NQ	NQ	NQ	NQ
	**%EE**	45.11	44.72	45.58	43.57	49.26	37.67
Total iridoids	**Encapsulated**	**463±14**	**378±24**	**578±18**	**228±15**	**601±19**	**347±13**
	**%EE**	**75.81**	**74.07**	**66.61**	**78.71**	**73.15**	**73.59**

Compound		7	8	9	10	11	12
Luteolin-7 diglucuronide	**Encapsulated**	160±3	150±6	202±5	107±4	198±5	139±3
	**%EE**	99.68	99.67	100.00	99.66	99.76	99.69
Apigenin-7-diglucuronide	**Encapsulated**	26.4±0.4	24±1	42±1	12.5±0.2	43±2	20±1
	**%EE**	100.00	100.00	100.00	100.00	100.00	100.00
Chrysoeriol-7-diglucuronide	**Encapsulated**	256±6	253±4	277±6	201±7	285±3	226±4
	**%EE**	99.01	99.10	99.30	99.01	99.17	98.95
Acacenin-7-diglucuronide	**Encapsulated**	121±3	114±5	148±2	83±2	151±5	101±4
	**%EE**	99.28	99.15	99.45	99.03	99.37	99.02
Methylquercetin	**Encapsulated**	NQ	NQ	NQ	NQ	NQ	NQ
	**%EE**	56.36	52.82	52.43	56.24	55.28	55.16
Dimethyl kaempferol	**Encapsulated**	8.0±0.1	5.2±0.2	16.5±0.3	0.633±0.003	16.9±1.5	5.3±0.2
	**%EE**	52.72	44.42	47.43	54.62	51.58	52.89
Dimethyl quercetin	**Encapsulated**	64±4	53±1	85±4	33±3	81±1	48±3
	**%EE**	57.60	52.07	54.85	56.63	56.44	54.36
Total Flavonoids	**Encapsulated**	**636±17**	**598±17**	**771±18**	**437±16**	**776±17**	**539±16**
	**%EE**	**86.38**	**84.33**	**83.67**	**87.77**	**85.19**	**85.78**
Verbasoside	**Encapsulated**	280±5	272±16	290±11	186±4	307±15	235±11
	**%EE**	86.10	86.41	76.09	87.84	82.57	83.49
Cistanoside F	**Encapsulated**	157±5	144±10	195±3	98±5	202±5	126±5
	**%EE**	89.06	89.06	82.40	90.56	87.06	86.81
β-Hydroxyverbascoside derivative	**Encapsulated**	18.7±0.3	17.6±0.5	28±1	10.3±0.4	27±1	14±1
	**%EE**	85.92	88.89	78.10	89.58	85.52	83.85
β-Hydroxyisoverbascoside derivative	**Encapsulated**	35±1	33±2	50.5±0.5	21.7±0.3	51±1	30±1
	**%EE**	89.48	90.10	82.08	92.63	87.11	87.81
β-Hydroxyverbascoside	**Encapsulated**	87±1	83±2	126±1	58±2	120±1	75±2
	**%EE**	86.24	86.78	76.23	89.08	81.38	82.48
β-Hydroxyisoverbascoside	**Encapsulated**	85±1	81±3	108±1	56.2±0.2	112±7	72±4
	**%EE**	85.67	85.35	74.79	88.51	80.24	83.35

Compound		**7**	**8**	**9**	**10**	**11**	**12**
Oxoverbascoside	**Encapsulated**	5.95±0.03	4.59±0.03	11.0±0.5	0.77±0.01	14±1	3.8±0.2
	**%EE**	85.83	86.59	77.64	86.18	83.92	80.46
Campneoside I	**Encapsulated**	0.62±0.01	0.64±0.01	4.2±0.1	NQ	3.7±0.3	0.76±0.03
	**%EE**	84.65	86.06	78.58	85.67	88.60	81.12
Verbascoside	**Encapsulated**	1380±33	1355±13	1841±15	1043±8	1814±11	1275±8
	**%EE**	73.79	74.87	65.34	75.79	70.65	69.26
Lariciresinol glucopyranoside	**Encapsulated**	44±2	35±1	61±1	23±2	54±1	34±1
	**%EE**	67.82	64.34	60.48	67.96	65.16	63.02
Verbascoside A	**Encapsulated**	38±1	31±2	53±4	28±1	43±1	33.7±0.3
	**%EE**	71.21	70.32	67.69	74.40	67.53	66.71
Isoverbascoside	**Encapsulated**	428±3	407±14	713±18	248±9	680±6	421±6
	**%EE**	73.99	75.53	66.10	75.39	71.40	70.61
Forsythoside A	**Encapsulated**	139±4	111±6	162±3	122.06±0.04	163±9	100±2
	**%EE**	56.31	50.82	52.06	55.67	65.11	61.74
Lipedoside A I	**Encapsulated**	16.3±0.8	15.6±0.8	24±1	9.6±0.1	23±1	12.1±0.5
	**%EE**	73.10	74.11	65.01	76.27	70.41	68.14
Leucoseptoside A	**Encapsulated**	188±4	181±5	239±5	132±3	246±10	163±8
	**%EE**	72.83	74.75	65.04	75.22	70.71	68.25
Martynoside	**Encapsulated**	118±2	121±5	161±2	81±1	168±8	99±2
	**%EE**	65.23	67.47	60.10	64.95	65.96	57.36
Martynoside isomer	**Encapsulated**	37.0±0.4	36.4±0.1	55±1	23.4±0.3	61.6±0.4	29±1
	**%EE**	65.47	66.64	61.65	64.22	69.09	56.42
Osmanthisude B	**Encapsulated**	53±3	48±1	72±2	35±1	78±4	40.6±0.3
	**%EE**	56.66	56.50	54.21	57.42	59.59	48.85
Total Phenylpropanoids	**Encapsulated**	**3110±67**	**2977±81**	**4195±70**	**2176±36**	**4166±85**	**2765±52**
	**%EE**	**73.93**	**75.02**	**65.78**	**75.59**	**71.22**	**69.85**
Total polar compounds	**Encapsulated**	4209±98	3953±122	5544±106	2841±67	5002±121	3651±81
	**%EE**	75.22	75.85	67.29	76.83	72.56	71.40

*NQ: Compound detected but its concentration is between detection and quantitation limits.

Table S6: Recovery of compounds from MD microparticles. Concentrations are exposed as µg of analyte/g of µparticle. Value = X ± SD.

Compound	Variable	1	2	3	4	5	6	7	8	9	10	11	12
Shanzhiside (Total fed: 101 ± 7)	%R	96.54	56.02	77.08	52.40	64.29	17.75	63.16	59.84	24.30	48.29	29.86	49.70
Gardoside (Total fed: 1330 ± 11)	%R	102.44	71.43	78.47	65.40	78.64	26.43	76.29	70.91	40.22	56.52	42.56	65.80
Ixoside (Total fed: 514.8 ± 0.5)	%R	84.98	52.92	61.20	50.85	55.49	16.00	56.29	48.46	21.88	43.01	28.09	44.87
Theveside (Total fed: 1360 ± 2)	%R	60.62	45.53	48.04	38.56	46.84	14.49	45.24	40.69	21.46	34.67	28.41	35.64
Myxopyroside (Total fed: 83 ± 4)	%R	65.34	46.25	46.06	47.35	48.89	17.04	47.99	43.04	23.68	35.27	30.50	38.72
Teucardoside (Total fed:44 ± 4)	%R	54.79	38.89	40.17	37.43	38.36	17.72	39.13	34.86	19.38	30.17	24.09	31.95
Lippioside II (Total fed: 43 ± 2)	%R	55.41	45.58	49.32	43.30	46.49	15.91	45.99	40.93	24.84	37.03	30.30	37.63
Lippioside I Derivative (Total fed: 18 ± 2)	%R	58.58	45.18	46.89	43.54	45.01	16.21	46.07	39.58	23.67	35.90	30.15	38.45
Lippioside I (Total fed: 36.2 ± 0.8)	%R	65.54	52.34	54.22	49.05	53.44	17.96	52.81	46.28	27.24	39.04	32.71	41.92
Hydroxycampsiside (Total fed: 22.93 ± 0.02)	%R	65.92	52.03	53.65	49.48	53.00	17.87	51.39	47.11	28.20	41.53	36.86	46.00
Lippianoside B (Total fed: 4.5 ± 0.3)	%R	61.98	56.87	65.76	57.54	61.89	21.53	58.92	51.51	32.53	45.07	34.59	37.08
Durantoside I (Total fed: 69 ± 4)	%R	62.94	56.04	57.05	53.19	56.40	19.18	54.24	48.17	29.20	43.49	35.77	46.12
Manuleoside H (Total fed: NQ)	%R	81.89	62.66	74.06	58.84	64.28	21.34	65.07	59.04	32.43	51.98	39.74	55.07

**Total iridoids (Total fed: 3626 ± 38)**	**%R**	76.05	54.67	59.98	50.48	57.90	18.96	56.70	51.26	27.88	43.13	33.20	46.45

**Compound**	**Variable**	**1**	**2**	**3**	**4**	**5**	**6**	**7**	**8**	**9**	**10**	**11**	**12**
Luteolin-7 diglucuronide (Total fed: 1260 ± 80)	**%R**	58.39	44.41	53.69	42.83	44.59	14.02	46.43	43.78	21.35	39.69	26.82	39.02
Apigenin-7-diglucuronide (Total fed: 173.2 ± 0.3)	**%R**	56.83	46.49	58.60	45.40	47.35	16.07	48.30	45.52	25.14	38.49	32.33	38.90
Chrysoeriol-7-diglucuronide (Total fed: 2816 ± 51)	**%R**	48.97	33.72	43.67	32.94	33.87	9.19	34.37	34.03	13.76	33.62	18.04	29.23
Acacenin-7-diglucuronide (Total fed: 1046 ± 28)	**%R**	50.92	40.20	48.40	38.94	40.55	12.43	42.74	40.54	19.10	37.55	24.82	34.91
Methylquercetin (Total fed: 14 ± 2)	**%R**	60.13	44.24	47.70	42.14	49.03	16.00	41.25	43.63	23.74	32.67	27.92	38.38
Dimethyl kaempferol (Total fed: 289 ± 27)	**%R**	55.12	42.22	50.29	39.32	49.08	13.05	36.66	38.31	20.89	29.31	24.76	30.97
Dimethyl quercetin (Total fed: 1121 ± 37)	**%R**	57.64	42.74	49.07	37.29	43.82	13.41	40.10	38.43	19.93	30.05	23.69	32.20
**Total flavonoids (Total fed: 6719 ± 225)**	**%R**	53.62	39.62	48.24	37.48	40.62	12.03	39.59	38.46	18.24	34.30	22.88	32.99
Verbasoside (Total fed:1619 ± 3)	**%R**	95.46	69.19	84.29	68.41	74.48	22.03	73.35	71.37	32.08	59.55	39.81	61.02
Cistanoside F (Total fed: 868 ± 14)	**%R**	93.05	71.68	85.71	66.25	73.69	24.26	72.84	67.57	36.18	56.58	45.16	58.23
β-Hydroxyverbascoside derivative (Total fed: 124 ± 10)	**%R**	66.73	54.33	63.03	51.91	55.41	19.55	57.21	53.16	31.72	45.57	36.27	46.89
β-Hydroxyisoverbascoside derivative (Total fed: 225 ± 5)	**%R**	70.58	57.00	64.88	55.43	58.51	20.35	59.51	56.66	32.82	48.07	39.82	50.82
β-Hydroxyverbascoside (Total fed: 678 ± 17)	**%R**	65.49	54.12	61.32	52.93	55.95	20.67	54.09	52.03	32.33	44.74	36.71	47.08
β-Hydroxyisoverbascoside (Total fed: 661 ± 13)	**%R**	61.87	49.93	60.37	49.53	54.69	18.39	54.94	52.60	29.26	44.75	35.77	46.20

Oxoverbascoside (Total fed: 20.8 ± 0.8)	**%R**	67.36	59.92	64.64	57.42	59.85	21.51	61.46	54.84	35.01	45.37	47.24	52.43
**Compound**	**Variable**	**1**	**2**	**3**	**4**	**5**	**6**	**7**	**8**	**9**	**10**	**11**	**12**
Campneoside I (Total fed: 0.29 ± 0.01)	**%R**	62.64	46.70	57.83	46.97	49.98	17.15	47.55	47.08	27.76	47.47	29.94	48.53
Verbascoside (Total fed: 86735 ± 741)	**%R**	42.32	38.77	47.14	40.62	42.10	15.50	47.29	46.10	25.24	45.43	29.34	45.29
Lariciresinol glucopyranoside (Total fed: 570 ± 23)	**%R**	48.90	42.07	43.63	39.94	43.43	16.09	43.74	38.16	24.34	31.64	25.65	36.55
Verbascoside A (Total fed: 431 ± 8)	**%R**	58.35	45.43	51.93	44.52	48.19	15.85	46.73	39.99	24.66	43.22	26.27	43.43
Isoverbascoside (Total fed: 27158 ± 327)	**%R**	51.60	49.69	58.33	52.53	54.42	21.53	62.53	59.83	35.82	57.45	40.87	62.24
Forsythoside A (Total fed: 346 ± 16)	**%R**	49.83	47.17	55.32	49.25	51.70	17.01	61.73	43.13	31.02	55.53	37.75	59.72
Lipedoside A I (Total fed: 129 ± 6)	**%R**	74.30	61.19	68.90	58.78	61.24	20.71	59.40	57.14	33.00	49.84	37.52	50.05
Leucoseptoside A (Total fed: 1757 ± 37)	**%R**	65.07	52.67	62.84	50.60	54.74	18.12	54.69	51.39	28.72	46.44	34.51	48.64
Martynoside (Total fed: 1174 ± 52)	**%R**	69.90	57.45	67.88	54.95	58.07	20.24	57.16	56.78	31.22	50.14	37.55	53.15
Martynoside isomer (Total fed: 360 ± 10)	**%R**	69.38	58.10	66.11	54.24	57.32	21.08	58.22	56.59	32.65	50.36	40.68	53.06
Osmanthisude B (Total fed: 544 ± 15)	**%R**	83.10	61.61	74.51	59.02	66.00	21.90	63.96	59.42	32.90	53.93	40.73	56.55
**Total phenylpropanoids (Total fed: 123400 ± 1298)**	**%R**	47.63	43.3	51.99	45.15	46.91	17.38	52.36	50.61	28.38	49.18	32.86	50.02
**Total polar compounds (Total fed: 133745 ± 1561)**	**%R**	49.97	43.51	51.99	44.52	46.77	16.82	51.05	49.17	27.12	47.03	31.67	47.75

foods MDPI

© 2020 by the author. Licensee MDPI, Basel, Switzerland. This article is an open access article distributed under the terms and conditions of the Creative Commons Attribution (CC BY) license (http://creativecommons.org/licenses/by/4.0/).
